# A model-based design strategy to engineer miRNA-regulated detection systems

**DOI:** 10.3389/fsysb.2025.1601854

**Published:** 2025-08-14

**Authors:** Renske J. Verkuijlen, Robert W. Smith

**Affiliations:** Laboratory of Systems and Synthetic Biology, Wageningen University and Research, Wageningen, Netherlands

**Keywords:** feed-forward loops, toehold-mediated strand displacement, multi-objective optimisation, multiple sclerosis, miRNA, threshold detection, iGEM

## Abstract

miRNAs are promising diagnostic biomarkers. These small RNA molecules are always present in the human body but become dysregulated when a person develops certain diseases. Although the detection of these biomarkers in cell-free tests is ongoing work, current systems often focus solely on detecting the presence or absence of a specific miRNA, rather than the miRNAs concentration. Thus, these tests may miss relative changes in miRNA concentration when disease-induced dysregulation occurs. This work, part of the WUR iGEM 2024 project (miRADAR), aimed to address this gap by incorporating an miRNA concentration-dependent threshold mechanism in a cell-free diagnostic test. In this system, continuous miRNA input concentrations need to be converted into a binary output signal, classifying the miRNA concentration as healthy (no output signal) or indicative of disease (strong output signal). To aid the experimental engineering of the test, here we use mathematical models to evaluate and assess different candidate networks. We apply a previously published multi-objective optimisation strategy to obtain designs that satisfy relevant constraints, such as low basal expression, high readout levels, and steep switching behaviour between low and high input miRNA concentrations. Models for three different biological mechanisms were compared based on their ability to generate the desired binary output signal. One approach used three-node protein networks (such as feed-forward loops), while the other two utilised RNA-based toehold systems. Overall, the toehold-mediated strand displacement systems demonstrated the most potential for experimental implementation. These systems are believed to be less burdensome in a cell-free environment, can be more readily engineered for new miRNA sequences, and showed high detection accuracy. Based on our results, we discuss how the inclusion of sequence-specific parameters could expand the design space of our mathematical models and how careful engineering of optimisation criteria is required to evaluate designs. Ultimately, our model-based study highlights that toehold-mediated strand displacement networks have the potential to be efficient miRNA detection systems for biosensing tools in the future.

## 1 Introduction

MicroRNAs (miRNAs) are a promising diagnostic marker and therapeutic agent. Research has identified numerous miRNAs with potential clinical applications in the detection and monitoring of neurodegenerative diseases such as Alzheimer’s and Parkinson’s disease (Gentile et al., 2022; Doroszkiewicz et al., 2022). The inhibition or activation of miRNAs involved in such diseases has been extensively studied as potential therapies (Walgrave et al., 2021; Nguyen et al., 2022). These small, single-stranded molecules are present in the body to regulate transcriptional gene expression (Lu and Clark, 2012). When carrying a disease, a person’s gene expression is differentially regulated and these changes, when compared to healthy controls, correlate with differential miRNA concentrations. This information can be utilised in a diagnostic tool, as measuring the change in concentration of disease-specific miRNAs can indicate the presence of that disease (Song et al., 2012; Wang and Zhang, 2020).

**FIGURE 1:**
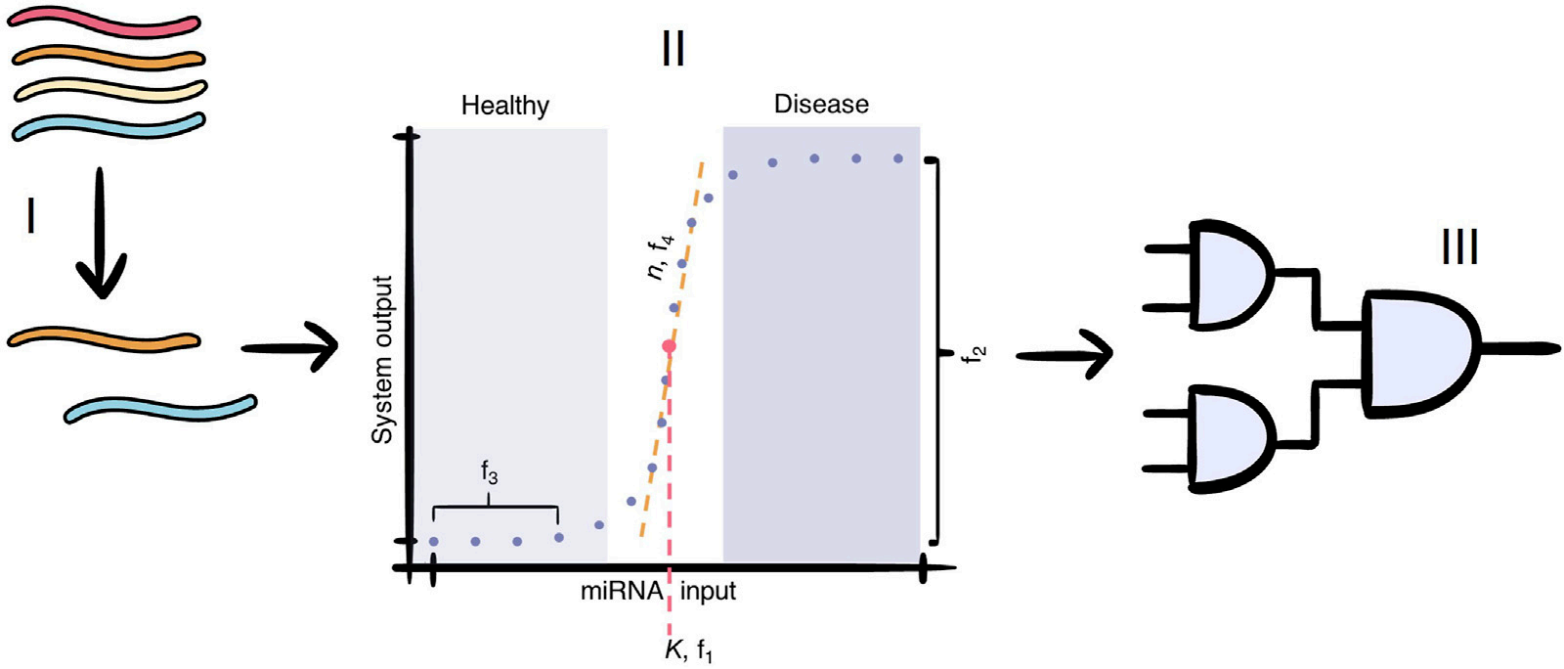
The cell-free paper-based miRNA test for multiple sclerosis consists of three main parts: I) the selection of miRNAs that are markers of MS; II) the conversion of the miRNA input concentration to a binary output signal, which differentiate healthy patients from those with MS. The dots represent the input concentrations at which our model evaluates the output dose-response curve. The values f schematically illustrate our scoring criteria for the models evaluated in this paper: $f_{1}$ evaluates the switching concentration of our system designs, $f_{2}$ evaluates the output concentration difference between low and high input concentrations, $f_{3}$ aims to measure (and reduce) basal output expression, and $f_{4}$ evaluates the steepness of the system’s switching behaviour. This concentration-dependent module is lacking in current cell-free paper-based miRNA test (this paper), and III) the modular detection module, which combines the binary signal of each miRNA into a single output signal.

This principle formed the basis of the WUR 2024 iGEM project, miRADAR, where miRNAs were used in a cell-free diagnostic tool to help detect the neurodegenerative disease multiple sclerosis (MS) (iGEM, 2024). In this disease, immune cells attack the myelin sheaths of the nerves, leading to a range of symptoms including muscle weakness and loss of vision (Ghasemi et al., 2017). The current diagnostic procedure, involving MRI scans and lumbar punctures, is invasive and believed to provide inconclusive results for 10%–30% of the patients [Christa Benit MD, personal communication; (iGEM, 2024; Tullman, 2013)]. At present, MS has no cure, but treatments delaying the degradation of the myelin sheaths and reducing symptom progression exist (Hauser and Cree, 2020). The earlier treatment is started, the better the quality of life of the patient can be conserved, demanding a timely diagnosis of MS (Giovannoni et al., 2016; Ziemssen et al., 2016). Previous studies have found multiple miRNAs dysregulated by MS, of which hsa-miR-484 and hsa-miR-145 are examples (Figure 1, step I) (Regev et al., 2018; Søndergaard et al., 2013). This emphasises that novel miRNA-based tests could be a valuable addition to the diagnostic procedure of MS.

In the miRADAR project, the WUR 2024 iGEM team envisioned creating a cell-free blood test to aid in diagnosing complex cases of MS (iGEM, 2024). To achieve this, a simple genetic detection circuit would need to be freeze-dried on paper discs. Upon the application of an MS-positive blood sample, the presence of several MS-specific miRNAs would trigger the genetic circuit and produce a colour marker that the patient and medical professionals can then observe. If the blood sample leads to a change in colour of the system’s output, then this suggests the presence of MS-specific miRNAs.

The key of the test lies in the concentration level of the MS-specific miRNAs; the miRNAs will always be present, but their concentration can be up- or downregulated in patients (Regev et al., 2018; Søndergaard et al., 2013; Ho et al., 2022). Current developments in other miRNA-based cell-free tests either do not take this into account and focus solely on detecting the presence of a specific miRNA, or depend on a difference in visual output, which is not sensitive enough when multiple miRNAs are detected in a single test (Li et al., 2019; Wang et al., 2023). Therefore, the addition of a concentration-dependent module is an important next step. Ideally, this module would give a binary output, where either the miRNA concentration is classified as healthy or indicative of the disease (Figure 1, step II). This signal conversion could be achieved by implementing a threshold mechanism, which distinguishes whether a miRNA is below or above a threshold concentration associated with healthy patients. No system output is generated when the input miRNA concentration is considered healthy (below threshold), while a large increase in system output is generated when the input miRNA concentration is considered indicative of MS (above threshold). The sharper this switch is, the more accurate the miRNA-based diagnostic test will be. Afterwards, individual binary signals can be integrated into a modular detection module that produces a single output signal allowing for the detection of multiple different miRNAs in a single test (Figure 1, step III). Two biological mechanisms that have the potential to create the desired threshold in the dose-response curve: i) a protein-based feed-forward loop (FFL) and ii) RNA-based toehold-mediated strand displacement (TMSD) systems.

### 1.1 Protein-based networks: feed-forward loops

**FIGURE 2:**
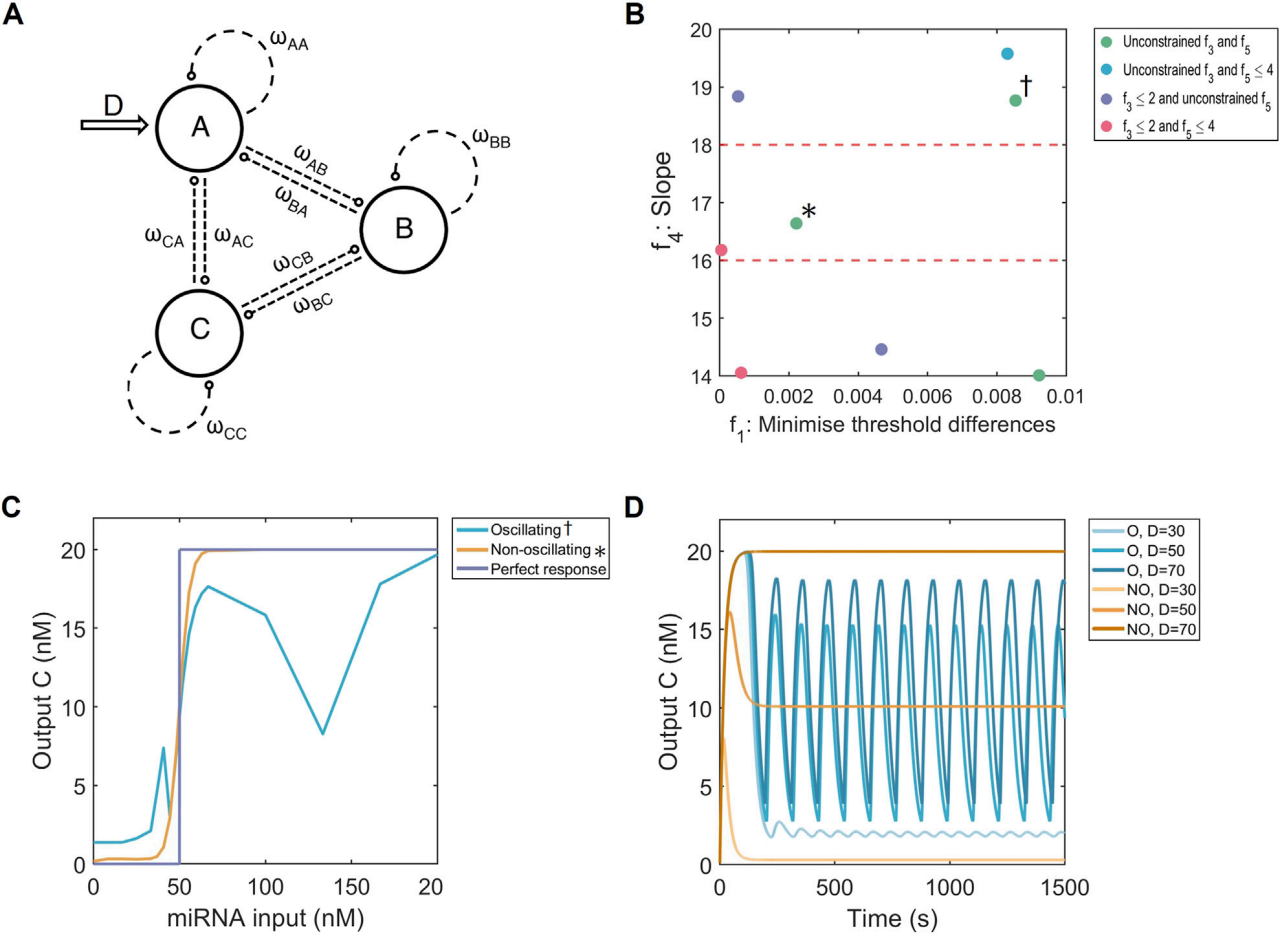
Multi-objective optimisation of the FFL system. **(A)** Hypergraph representing three-node networks with all possible connections. The input is on node A (D, arrow), while C is regarded as the output of the system. $\omega_{ij}$ represents the strength and type of node connection. Image adapted from (Otero-Muras and Banga, 2017). **(B)** Multi-dimensional results of the optimisation strategy displaying the threshold distances ( $f_{1}$ ; absolute difference from $K_{\mathrm{expected}}$ of 50 nM) and the three highest slope intervals ( $f_{4}$ ; n from Hill function). The solutions are coloured according to their constraints on $f_{3}$ (basal expression) and $f_{5}$ (number of node connections). Red dashed lines show slope optimisation boundaries. ∗ : non-oscillating solution. † : oscillating solution. **(C)** Dose-response curves for an FFL causing oscillations (blue) and one without (orange), and a perfect binary response (purple). $K_{\mathrm{expected}}$ = 50 nM. **(D)** Time responses for an oscillating system (blue, O) and non-oscillating system (orange, NO) for 3 doses D : one below (30 nM), one at (50 nM) and one above (70 nm) the $K_{\mathrm{expected}}$ of 50 nM.

Many genetic circuits found in nature contain core network motifs consisting of a limited number of components (Milo et al., 2002). One example of such a network motif are feed-forward loops (FFLs) where three nodes can interact (in)directly with each other through activation and inhibition [Figure 2A; (Alon, 2007)]. FFL nodes can consist of an interplay between transcription factors, proteins, DNA, and RNA, and are consistently found to regulate processes like adaptation, noise filtering, biphasic behaviour, and oscillations (Ma et al., 2009; Pieters et al., 2021; Kim et al., 2008; Zhang et al., 2016). The basic structure of an FFL consists of a direct interaction between input node A and output node C, combined with an indirect reaction through node B. The FFL is called coherent if the direct effect of node A on node C and the indirect effect through node B are consistent. An example of a coherent network is that A activates C directly, but also activates B which also activates C in turn: in this case, A has a net positive effect on C via both paths. If the effect through both pathways is antagonistic, the FFL is considered incoherent. Next to these main reactions from node A to node C, additional regulation between the nodes is possible, including autoregulation and feedback which allows for more complex behaviour to emerge (Alon, 2007; Mangan and Alon, 2003).

Previous research has shown that such networks may be relevant for our miRNA detection system. While looking for networks that show adaptation using three-node networks, (Ma et al., 2009), also found topologies capable of producing binary outputs (C in Figure 2A) given input concentrations (D in Figure 2A) below and above some threshold. A three-node coherent FFL has also been shown in (Rahman et al., 2018) to create a dose-response curve with threshold-like behaviour. Furthermore, through modelling T-cell receptor binding, an incoherent FFL was found to be the basis of larger networks capable of producing binary output responses (Lever et al., 2016). Consequently, based on these results, we hypothesise that transcription factor-based FFLs are a good candidate for the genetic network required within the miRADAR cell-free test.

### 1.2 RNA-based networks: toehold-mediated strand displacement

The goal of the miRADAR project was to develop a cell-free paper-based test, which increases accessibility for patients. To create an efficient cell-free system, we are required to limit the number of biological components needed to produce an output. As we envision that our FFL systems consist of transcription and translation of node components, our cell-free test will also require compounds to process DNA and RNA into protein which will accelerate energy usage and could limit output responses (Stögbauer et al., 2012). To combat this issue, a more energy-efficient system based solely on base-pair binding called toehold-mediated strand displacement (TMSD) could prove useful. At the core of TMSD lies a double-stranded RNA complex, with a free annealing region called the toehold (Zhang and Winfree, 2009). An input RNA with a complementary domain to the toehold and the rest of the RNA strand will anneal to the toehold and displace the first-bound RNA strand, as its hybridisation energy is higher. In a kinetic model, this TMSD reaction can be simplified into one rate because the initial toehold annealing is the rate-limiting step (Qian and Winfree, 2011; Akay et al., 2024). The sequence design determines which reaction will proceed at which rate, enabling engineering of the mechanism to desired needs (Zhang and Seelig, 2011). With the addition of other strands, behaving like inhibitors or catalysts called fuels, the TMSD reaction can be expanded to perform multiple functions (Qian and Winfree, 2011; Chen et al., 2023). Here, two variants of TMSD will be tested, namely i) with fuel reactions (TMSD-F) and ii) without fuel reactions (TMSD-NF).

The TMSD system has previously been integrated into miRNA diagnostic tests as an amplification module (Liang et al., 2020; Zhang et al., 2020). The miRNA concentrations can be measured, but this TMSD system was not sensitive enough to detect small dysregulations in miRNA concentration levels in the proposed MS test (Lee et al., 2021). Previous studies illustrated the potential of TMSD-based systems to produce binary system outputs that differentiate between low- and high concentrations of miRNA (Qian and Winfree, 2011; Seelig et al., 2006). However, both approaches do not generate a sharp enough switch and suffer from high basal expression, which impairs the quality of the binary signal.

**FIGURE 3:**
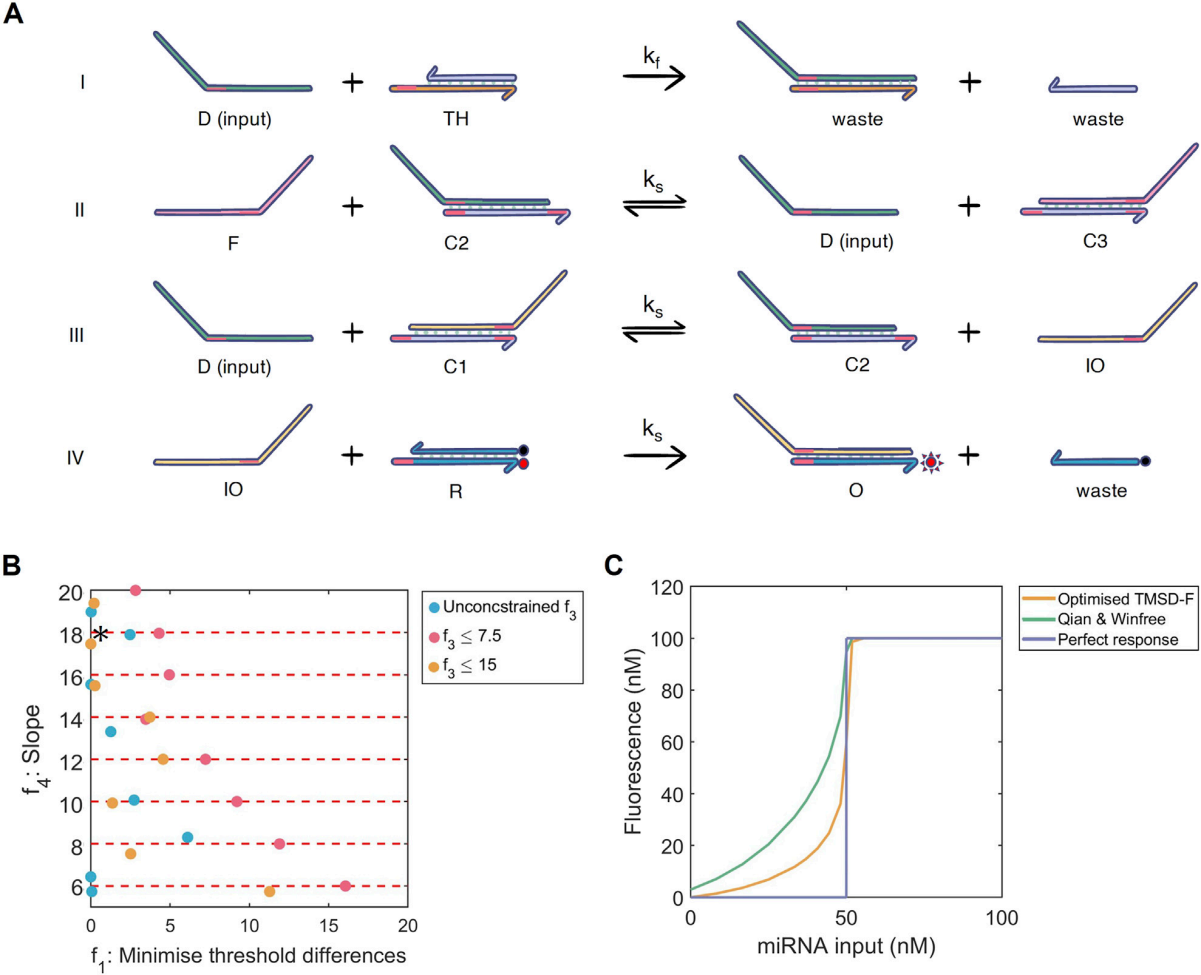
**(A)** Multi-objective optimisation of the toehold-mediated strand displacement with fuel (TMSD-F) system. I) The threshold reaction: input strand D anneals to the threshold strand TH to produce a waste strand W . II) The fuel reaction: the fuel strand F releases input D from C2 to produce D and C3 . III) The TMSD reaction: input strand D anneals to C1 to form C2 and intermediate output IO . IV) The reporter reaction: intermediate output IO displaces the quencher R resulting in fluorescence O and a waste strand W . With a longer toehold, reaction I can proceed at a faster kinetic rate $\left(k_{f}\right)$ than reactions II-IV (all $k_{s}$ ). The universal toehold is shown in pink. Image adapted from (Qian and Winfree, 2011) **(B)** Multi-dimensional results for the TMSD-F optimisation displaying the threshold accuracy ( $f_{1}$ ; absolute difference from $K_{\mathrm{expected}}$ ) and slope ( $f_{4}$ ; n from Hill function). The solutions are coloured according to their additional constraint on $f_{3}$ (basal expression). $K_{\mathrm{expected}}$ = 50 nM. Red dashed lines show slope optimisation boundaries. ∗ : best-performing solution. **(C)** Dose-response curves of the best-performing TMSD-F (orange), unoptimised TMSD-F [green, (Qian and Winfree, 2011)] and a perfect binary response for $K_{\mathrm{expected}}$ = 50 nM (purple).

To improve on the initial TMSD system, (Qian and Winfree, 2011), added two new reactions. We will refer to this system as TMSD-F (Figure 3A). The first reaction utilised miRNAs with antisense sequences to the input miRNA in order to compete with the TMSD reactions and prevent the system responding until sufficient input miRNA was present. The second additional reaction, referred to as the fuel reaction, created a positive feedback mechanism whereby input miRNAs could repeatedly trigger the TMSD gate. These additions allowed the system to approach binary output responses given different input miRNA concentrations. Furthermore, all the reactions within this expanded TMSD system make use of the same toehold sequence, referred to as the universal toehold. The universal toehold increases modularity and allows for multiple TMSD reactions to be linked to each other, but it also generates side reactions that have an unknown influence on the quality of the resulting output dose-response curve.

**FIGURE 4:**
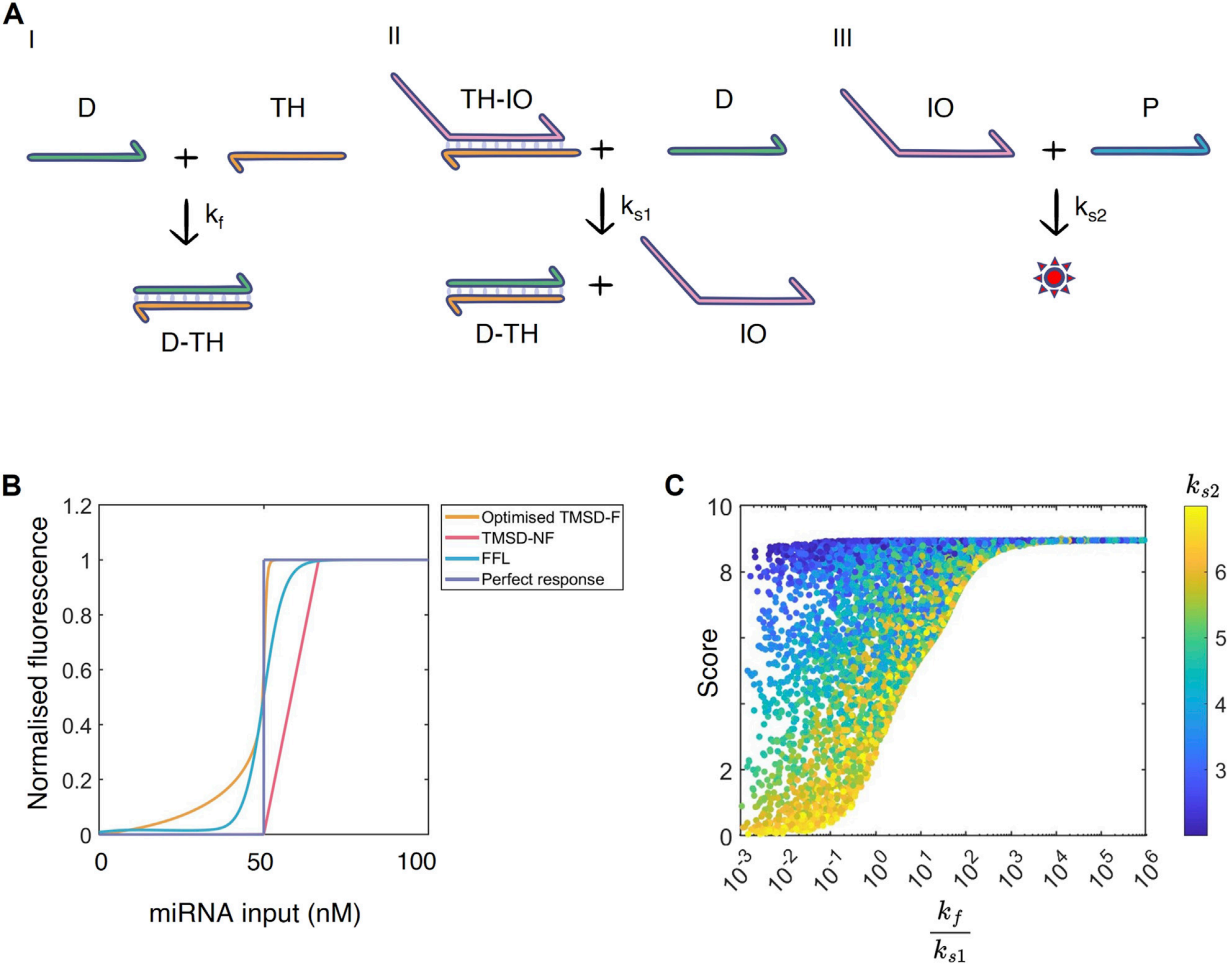
**(A)** The TMSD-NF system analysed by the WUR 2024 iGEM team. I) The threshold reaction: input D anneals to threshold strand TH to form a complex D-TH . II) The TMSD reaction: D binds to TH-IO to release intermediate output IO and D-TH . III) Aptamer reaction: aptamer P anneals to intermediate output IO to produce fluorescence O (denoted by a star). The toehold is shown in orange. **(B)** Normalised dose-response curves for the FFL (blue), TMSD-F (orange), TMSD-NF systems (pink) and a perfect binary response (purple). The curves were normalised by dividing the fluorescent output for each dose by the maximum system output. $K_{\mathrm{expected}}$ = 50 nM. **(C)** Parameter space exploration of the TMSD-NF system. The $\frac{k_{f}}{k_{s1}}$ ratio (log scale) is plotted against the score (maximum of 10). The colour bar represents the $log_{10}\left(k_{s2}\right)$ .

The miRADAR project also considered a further simplification of the TMSD-F system (iGEM, 2024). This new system removed the fuel component and the universal toeholds. Consequently, we named the system TMSD-NF (Figure 4A).

### 1.3 Multi-objective optimisation algorithms for model design

FFL and TMSD are biological mechanisms that could, with further optimisation, form a sharp threshold in the dose-response curve. With the described kinetic models, we wish to optimise the parameters of our systems to increase the accuracy and steepness of the threshold while additionally lowering the basal expression before that threshold point (Figure 1, step II, see a schematic of these behaviours labelled $f_{1}$ - $f_{4}$ ). Previous research has varied parameters of FFL networks to observe how parameter relationships impact FFL performance (Ma et al., 2009). However, an exhaustive search, as used in (Ma et al., 2009), can be extremely computationally demanding. Therefore, the mixed-integer multi-objective optimisation framework laid out by (Otero-Muras and Banga, 2017) is a good fit to efficiently search the model design space. By tackling the problem as an optimisation problem instead, FFL networks were able to show behaviour like adaptation, oscillation, and fold-change detection (Otero-Muras and Banga, 2017; Otero-Muras and Banga, 2016). The wide range of applications indicates that an FFL engineered to produce a binary response should be possible.

In multi-objective optimisation problems, trade-offs between different objectives are likely. As an example, one could envision that, for the threshold mechanisms, a higher slope (i.e., the sharpness of the increase between “healthy” and “disease” in Figure 1, step II, $f_{4}$ ) would reduce the threshold accuracy (i.e., is the lowest output-producing input concentration close to the desired switching/threshold concentration; $f_{1}$ in Figure 1, step II). The algorithm from (Otero-Muras and Banga, 2017) does not search for the sole most optimal solution in the complete objective space but finds the best solution in a specific part of one objective space. Essentially, the multi-objective optimisation is transformed into multiple single-objective optimisations. This method is also known as the ε -strategy, which has been applied to various multi-objective optimisation problems, including ones outside the biological domain (Du et al., 2014; Elmi et al., 2023). Given the slope and threshold accuracy example above, the slope objective space can be split into multiple constrained intervals. Inside each slope interval, the difference between simulated and desired threshold is minimised with a local optimisation routine. Finally, all the results can be combined such that the whole objective space is visualised by plotting the different objective values against each other. This way, it is possible to find the trade-off these two objectives might have, which helps to engineer the systems further. From these results, we can infer which network characteristics are important for our desired system behaviour (Mavrotas, 2009). Such concepts are related to Pareto fronts or Pareto optimality that have been utilised before in biological engineering problems (Boada et al., 2016; Taneda, 2015; Szekely et al., 2013).

By applying the above-described optimisation strategy of (Otero-Muras and Banga, 2017), in this work we will optimise a protein-based FFL network and our two RNA-based TMSD systems (TMSD-F and TMSD-NF) to increase their functionality as threshold mechanisms. We will go on to show that all three network designs have the potential to produce near-binary output signals in response to different input miRNA concentrations. These results will show that TMSD-based networks outperform FFLs in their ability to respond to different miRNA inputs, and we will discuss how TMSD networks could be further engineered in the future. These findings advance the study of miRNAs as a diagnostic tool by exploring the necessary concentration-dependent module that current tests lack.

## 2 Methods

### 2.1 Mathematical models

#### 2.1.1 Three-node networks and feed forward loops

The feed forward loop (FFL) system consists of three nodes, which can inhibit or activate each other and themselves (Figure 2A). We assume these connections to represent transcription and translation where one node’s mRNA is translated to a transcription factor that can regulate preceeding nodes. A single node connection, denoted ω , in our three-node networks can be described by combining the regulation type y and regulation strength x into ω $\left(\omega_{ij}=x_{ij}y_{ij}\right)$ , where node i acts on node j (Otero-Muras and Banga, 2017). The regulation type y is an integer with three possible values: 1 for activation, −1 for inhibition and 0 for no effect. The regulation strength x can take any real positive number, with higher numbers reflecting a higher effect. The three-node network is represented as a combination of all the possible ω ’s. Importantly, we are not restricting or enforcing the FFL structure on our resulting three-node networks with the hope that we may uncover previously studied networks from our analysis (as in Ma et al., 2009). The dynamics over time for one node — A , B , and C — are modelled as an ordinary differential equation, which incorporates the necessary ω ’s into the equations. Each ODE consists of a Michaelis-Menten-style production term, whereby production is inhibited by proteins within the network, and a linear degradation term. The input miRNA, D (Figure 2A), regulates production of A and is assumed constant over time since the half-life of many miRNAs is longer than the timeframe of our systems [estimated to be approx. 16 h in (Wang and Liu, 2022)]. The complete set of model equations are in Supplementary Methods S1.3.

The ODEs were solved with a CVODES solver provided by Serban and Hindmash which was adjusted by Otero-Muras and Banga (2017) to accommodate mixed-integer parameters (Otero-Muras and Banga, 2017; Serban and Hindmarsh, 2005). The absolute and relative tolerance were defined as $10^{-12}$ and the maximum step size was set to infinity as specified by Otero-Muras and Banga (2017).

#### 2.1.2 Fuel-regulated toehold mediated strand displacement system

The ODEs for toehold mediated strand displacement system with a fuel reaction (TMSD-F) were derived by Qian and Winfree (2011) and based on the law of mass action. The system has two kinetic rates: a fast kinetic rate $\left(k_{f}\right)$ and a slow kinetic rate $\left(k_{s}\right)$ , and we assume that all fast or slow reactions proceed at the same rate. The threshold reaction (Figure 3A, I has to take place quickly (with rate $k_{f}$ ) as the input miRNA, D , needs to bind with the threshold sequence, TH , before the TMSD reaction takes place and D is used to produce an output, O (Figure 3A, III). The faster kinetic rate occurs as a result of designing a longer toehold to initiate the reaction, which increases the hybridisation energy (Zhang and Seelig, 2011). The addition of a fuel component, F , allows for the re-use of input miRNA sequences, D , to produce output, O . The fuel, TMSD, and reporter reaction are initiated by the same toehold sequence, referred to as the universal toehold (Figure 3A, II–IV). As the same sequence is used, we assume that these reactions proceed at the same rate $k_{s}$ (Qian and Winfree, 2011; Akay et al., 2024). These four main reactions are accompanied by side reactions (denoted waste sequences, W ) caused by the universal toehold and a leakiness reaction, where the fuel strand directly produces intermediate output. Using the law of mass action, the reactions presented in Supplementary Methods S1.4 can be translated into ODEs for the input miRNA D , threshold sequence TH , the waste sequence W , the TMSD sequences C1 , C2 , and C3 , the intermediate output IO , the reporter R , and the output O .

The ODEs were solved with the MATLAB CVODES stiff solver under standard parameters, except absolute tolerance = $10^{-6}$ and relative tolerance = $10^{-6}$ so solutions with high accuracy could be achieved (Gardner et al., 2022; Hin et al., 2005). With the kinetic rates determined from data by Qian and Winfree (2011) (Supplementary Table S3), our model reached steady state in 2,500 s (Supplementary Figure S11) which is a similar time-frame as the original publication. Consequently, we measured our simulated output at this timepoint during the optimisation process (Supplementary Figure S11).

#### 2.1.3 Toehold mediated strand displacement systems without fuel

The ODEs for the fuel-removed TMSD system (TMSD-NF) were adapted from TMSD-F and consist of an equation for the threshold reaction (with rate $k_{f}$ ), the TMSD reaction (with rate $k_{s1}$ ) and the binding to a fluorescent aptamer (with rate $k_{s2}$ ; Figure 4A). The complete set of model reactions and equations are in Supplementary Methods S1.5. The ODEs describe the change over time of the input miRNA D , the threshold sequence TH , the resulting complexes formed with the intermediate output IO and the output sequence P .

The ODEs were solved with the MATLAB CVODES non-stiff solver using standard parameters, except absolute tolerance = $10^{-6}$ and relative tolerance = $10^{-6}$ (Gardner et al., 2022; Hin et al., 2005).

### 2.2 Mathematical representation of a dose-response curve

Mathematically, dose-response curves can be described with the Hill function

$$O=I+O_{\max}\frac{D^{n}}{K_{\mathrm{Hill}}^{n}+D^{n}},$$ (1)

where $K_{\mathrm{Hill}}$ is the concentration of input miRNA D that results in half of the differential system output O (Ang et al., 2013), i.e. if $K_{\mathrm{Hill}}=D$ then the system reaches half the maximal output possible $\left(I+O_{\max}/2\right)$ where the intercept I is the system output O at D=0 . The slope of the curve is denoted by n . The ODEs of each system had to be simulated for multiple input doses of miRNA D to generate a dose-response curve from which the parameters of the Hill function could be extrapolated and used in scoring functions.

Simulating a large number of doses would slow down the optimisation drastically, so a minimal amount of doses, which still accurately represent the curve, had to be determined (Supplementary Methods S1.1). In total, 19 doses are necessary to create an accurate dose-response curve, with 10 of the total doses centred around the threshold dose. This is shown schematically in Figure 1 (step II).

### 2.3 Optimisation objectives

The objectives of the optimisation of the FFL and TMSD-F systems are based on the desired dose-response curve (Figure 1, step II). Ideally, this curve has i) a high threshold accuracy, ii) a high system output after the threshold is passed, iii) a low basal expression before the threshold, and iv) a steep slope.

The K from the Hill function (Equation 1) should be at the dose where the miRNA-dependent threshold should be. Therefore we want to minimise the difference between the threshold concentration we want $\left(K_{\mathrm{expected}}\right)$ with the threshold concentration we obtain with our model $\left(K_{\mathrm{Hill}}\right)$ . We define our first objective to minimise this difference following

$$f_{1}\left(K_{\mathrm{Hill}}\right)≔min\;\left(K_{\mathrm{Hill}}-K_{\mathrm{expected}}\right),$$ (2)

where $K_{\mathrm{Hill}}$ is calculated with the Hill function obtained from an optimisation solution and $K_{\mathrm{expected}}$ is the desired threshold value. This objective was minimised in the optimisation, and we will refer to this as minimising the threshold differences or maximising threshold accuracy. The other three objectives were set as optimisation constraints, meaning that objectives $f_{2}$ , $f_{3}$ and $f_{4}$ are constrained to user-input values that comply with well-performing dose-response curves.

The second objective, which assesses the maximum system output can be defined as constraint

$$f_{2}\left(O\left(D\right)\right)≔l_{1}<log_{10}\left(O\left(D_{\mathrm{end}}\right)-O\left(D_{1}\right)\right)<u_{1},$$ (3)

where the lower boundary $l_{1}$ and the upper boundary $u_{1}$ were set to reach the known maximum system output. For FFL the boundaries were set to $l_{1}=1$ and $u_{1}=1.5$ , while for TMSD-F $u_{1}=2.5$ . By defining $l_{1}\geq 1$ then we are enforcing our output at $D_{\mathrm{end}}$ , $O\left(D_{\mathrm{end}}\right)$ , to be greater or equal to the output produced at the lowest input concentration, $O\left(D_{1}\right)$ . Next, the basal expression was quantified by summing up the system outputs induced by the four lowest doses of input miRNA

$$f_{3}\left(O\left(D\right)\right)≔l_{2}<\overset{4}{\underset{i=1}{\sum }}O\left(D_{i}\right)<u_{2},$$ (4)

where i represents the index of the dose D and both $l_{2}$ and $u_{2}$ are a user-input value to constrain the basal expression. In this work, $l_{2}$ is always set to 0, while $u_{2}$ is set to 2 for the FFL optimisation and 7.5 or 15 for the TMSD-F optimisation. The decision to use the four lowest input doses is discussed in Supplementary Methods S1.1, 1.2 where we show that simulating more input doses increases computational time to optimise a single model, whilst including more doses within this scoring function does not influence the score per data point for an optimal model.

According to (Otero-Muras and Banga, 2017), the slope objective was split into multiple interval constraints. This objective function was consequently defined as constraint

$$f_{4}\left(n\right)≔l_{3}<n<u_{3},$$ (5)

where the Hill function was used to calculate slope n , which had to remain inside the interval boundaries $l_{3}$ and $u_{3}$ for a solution to be accepted. The values for $l_{3}$ and $u_{3}$ are iteratively updated, so $f_{1}$ was separately optimised for each interval. In this work, we optimised the system 10 times for 10 evenly spaced intervals between 0 and 20. We assume that values of n above 20 are not biologically feasible and higher values do not lead to much improvement in the dose-response curve. The first single objective optimisation thus minimised $f_{1}$ , while $f_{4}$ was kept between a value of 0 and 2. The second optimisation then aimed to minimise $f_{1}$ with $f_{4}$ constrained to bounds of 2 and 4, and so on.

In the optimisation of FFL and three-node networks, a fifth constraint objective was added to limit the number of node connections as follows

$$f_{5}\left(y\right)≔l_{4}<\underset{i,j}{\sum }|y_{i,j}|<u_{4},$$ (6)

with i=A,B,C and j=A,B,C resulting in a summation of 9 absolute y (regulation effect) values in total. The bounds $l_{4}$ and $u_{4}$ are user-input integers and were defined here as $l_{4}=0$ and $u_{4}=4$ to limit the total connections in our three-node system to a maximum of 4. In a three-node network the maximum amount of node connections is 9 and we do not enforce the FFL structure on our networks.

### 2.4 Multi-objective optimisation algorithm

The optimisation algorithm combined the global solver eSS (enhanced scatter search; release 2010A) and the local solver misqp (for FFLs and three-node networks; version 7.1) or fmincon (for TMSD systems; version MATLAB 2024a) into a hybrid solver (Otero-Muras and Banga, 2017; Egea et al., 2009; Exler and Schittkowski, 2007). After 10 iterations of the global solver, the local solver refined the best solution. This process was repeated until a total of 10,000 evaluations were completed. In the global solver, 320 diverse solutions were initially generated. From these, 20 solutions were put in the reference set, whose values were iteratively updated to the 20 new best solutions (Otero-Muras and Banga, 2017). All solver parameters were obtained from (Otero-Muras and Banga, 2017).

### 2.5 Latin hypercube sampling for parameter space exploration of TMSD-NF

The TMSD-NF system consists of three reactions rates ( $k_{f},k_{s1}$ , and $k_{s2}$ ). This allows us to more extensively assess the relationship between these parameters and system performance. To do this, we sampled permissible parameter space for the TMSD-NF system with a Latin Hypercube sample ( $n_{\mathrm{samples}}$ = 10,000). The objectives presented above were slightly adjusted to better fit the behaviour of the TMSD-NF system, which has linear dose-response curves that cannot be described by the Hill function.

The maximum output of the system is determined by the known concentration of aptamer P (Supplementary Methods S2.3.1). Therefore, we know what the expected maximum output $O_{\max}$ of the system should be $\left(O_{\mathrm{max,expected}}\right)$ . We, therefore, scored how close the observed maximum output is to the expected maximum as

$$s_{1}\left(O\right)≔O_{\mathrm{max,observed}}-O_{\mathrm{max,expected}}.$$ (7)

to score the basal expression before the threshold, the formula remained unadjusted from above, except the lower and upper boundaries were removed:

$$s_{2}\left(O\left(D\right)\right)≔\overset{4}{\underset{i=1}{\sum }}O\left(D_{i}\right).$$ (8)

to measure the steepness of TMSD-NF systems, we replaced function $f_{4}$ (Equation 5) with

$$s_{3}\left(O\left(D\right)\right)≔\int_{D=K_{\mathrm{expected}}}^{D_{\mathrm{end}}}\frac{d\;O\left(D\right)}{d\;D}dD.$$ (9)

this change was required since TMSD-NF systems produced optimal dose-response curves with sharp transitions between healthy and disease regimes (Figure 1, step II) once the threshold input dose has been crossed - a qualitatively different behaviour to which we had before. This means that fitting a Hill function, approximating n and using $f_{4}$ as a scoring metric, in these cases became unreliable in high-throughput since the concentration where the output was half the maximal value could not be fixed to $K_{\mathrm{expected}}$ . In the Supplementary Methods S1.2 we discuss the impact of this change and show that, for simulated test cases, the formulation of $s_{3}$ produces qualitatively the same results as function $f_{4}$ .

The change in gradient just before and after $K_{\mathrm{expected}}$ indicates the threshold accuracy, where a higher value correlates with minimal differences between the observed threshold and the expected threshold. The corresponding scoring function is defined as

$$s_{4}≔\frac{d\;O\left(D_{K_{\mathrm{expected}}-1}\right)}{d\;D_{K_{\mathrm{expected}}-1}}-\frac{d\;O\left(D_{K_{\mathrm{expected}}+1}\right)}{d\;D_{K_{\mathrm{expected}}+1}}.$$ (10)

The final score was computed as

$$Score=w_{1}s_{1,rescaled}+w_{2}s_{2,rescaled}+w_{3}s_{3,rescaled}+w_{4}s_{4,rescaled},$$ (11)

where $w_{1}=0.1$ and $w_{2}=w_{3}=w_{4}=0.3$ and $s_{n,rescaled}=l+\left[\frac{s_{n}-s_{n,min}}{s_{n,max}-s_{n,min}}\right]\left(u-l\right)$ . The lower boundary l of the rescaling was set to 0 and the upper boundary u of the rescaling was set to 10. $s_{n,min}$ and $s_{n,max}$ are the minimum and maximum values for each corresponding set of scoring function outcomes from our sampling. The rescaling to a standardised range of 0–10 was necessary for a fair comparability of the different scoring functions as they originally varied over different ranges. Without this, scoring functions with large outcome values would disproportionately influence the final score. The scoring function $s_{2}$ was reverse-coded to convert the lowest basal expressions to the highest scores. The scoring function $s_{1}$ was assigned a lower weight because it was consistently observed to achieve satisfactory values and thus less helpful in differentiating the curves.

## 3 Results

### 3.1 Optimising three-node networks and feed-forward loops

The first mechanism applied to convert continuous input concentrations of miRNA into a binary fluorescent signal are three-node networks, of which the FFL system is a special example (Figure 2A). In the multi-objective optimisation process (Section 2.3, Equations 2 - 6), the threshold accuracy $\left(f_{1}\right)$ , i.e. how close the $K_{\mathrm{Hill}}$ of the dose-response curve is to $K_{\mathrm{expected}}$ , was optimised under additional constraints on the basal expression $\left(f_{3}\right)$ , slope $\left(f_{4}\right)$ and the number of node connections in the network $\left(f_{5}\right)$ . The closer the value for $f_{1}$ is to 0, the smaller the difference is between the system’s value of K and what we wish to achieve. Conversely, this could be a considered as maximising the threshold accuracy. We visualise our results over a two-dimensional $f_{1}$ - $f_{4}$ sample space, i.e. the threshold difference is plotted against the slope from the estimated Hill function. Multiple intervals of slope values n , ranging from 0 to 20 in increments of 2, were tested, resulting in ten combinations of threshold accuracies $\left(f_{1}\right)$ and slope values $\left(f_{4}\right)$ . Based on our results, we observed a trade-off where higher slope values $\left(f_{4}\right)$ combine with lower threshold differences $\left(f_{1}\right)$ . The effect of limiting the basal expression $\left(f_{3}\right)$ or the number of node connections $\left(f_{5}\right)$ in our three-node networks was judged according to their effect on $f_{1}$ and $f_{4}$ .

According to the plotted search space, our three-node systems consistently reached minimal threshold differences, showing that high threshold accuracies are robust to changes in the slope of output dose-response Hill functions (Figure 2B; Supplementary Figure S4). These values were reached regardless of the limits set by $f_{3}$ and $f_{5}$ , indicating that low basal expressions and fewer node connections do not compromise the slope and threshold accuracy. In other words, it is possible to construct a three-node system complying with all the set objectives. Outliers with poor threshold accuracies exist but this was an issue for every constraint combination, suggesting that the algorithm might sometimes be stuck in a local minima (Supplementary Figure S4).

Based solely on the objective values, multiple well-performing systems exist but this is not reflected in the simulated dose-response curves. There, some curves show irregular behaviour, where the output at higher doses of input miRNA is not constant. As an example, from two systems performing similarly in our $f_{1}$ - $f_{4}$ search space, one dose-response curve shows irregular behaviour (Figures 2B,C, dagger), while the other does not (Figures 2B,C, asterisk). Their respective responses over time expose that systems with irregular dose-response curves do not reach steady states, but instead, oscillate (Figure 2D). The differences in behaviour are reflected in the topologies of the system (Supplementary Figure S6). Both non-oscillating and oscillating FFL mechanisms involve strong negative regulation on A (either through $\omega_{AA}$ , $\omega_{BA}$ or $\omega_{CA}$ ). Our non-oscillating systems tend to form more regular FFL systems or linear pathways, but oscillating systems form a Goodwin oscillator [positive $\omega_{AB}$ , positive $\omega_{BC}$ and negative $\omega_{CA}$ ; (Goodwin, 1965)]. Therefore, careful design of three-node networks to constrain our algorithm to FFL systems could thus be vital to prevent oscillations.

### 3.2 Optimising fuel-regulated toehold systems

The TMSD system solely relies on nucleotide binding, providing an advantage over the energy-demanding transcription and translation necessary for the functioning of an FFL. The TMSD-F system contains a fuel strand, which catalytically speeds up and increases the production of fluorescent output (Figure 3A II). The same objectives as for the three-node networks, except for $f_{5}$ , were applied to optimise the reaction rates and improve the dose-response curve produced with the TMSD-F system from (Qian and Winfree, 2011).

Basal expression limits ( $f_{3}$ , Equation 4) of 7.5 nM and 15 nM, alongside unconstrained $f_{3}$ , were used as constraints for TMSD-F optimisation. The effect on the threshold accuracy ( $f_{1}$ , Equation 2) and slope ( $f_{4}$ , Equation 5) were again evaluated over a two-dimensional space. A restriction of $f_{3}$ to 15 nM did not affect the threshold accuracy of the switch compared to unconstrained basal expression (overlap of orange and blue dots in Figure 3B). However, further reducing the basal expression to 7.5 nM detrimentally reduced the threshold accuracy, especially for lower slope values (red dots in Figure 3B). The plotted dose-response curves revealed that the curve shifts slightly to the right of the $K_{\mathrm{expected}}$ when $f_{3}$ was limited to 7.5 nM, which reduced the threshold accuracy (Supplementary Figure S7). In contrast to our three-node networks and FFL systems, no oscillations are observed in the dose-response curve.

From the observed search space, we discovered that constraining our slope n to be between 16 and 18 $\left(f_{4}\right)$ and the basal expression to be at most 15 nM $\left(f_{3}\right)$ provided a desirable solution for our purposes. The resulting network combines the minimal difference between simulated and expected K value, a high slope n , and a limit on the basal expression (Figure 3B, asterisk). Compared to (Qian and Winfree, 2011), the optimised system had a slower $k_{s}$ rate, which slows down the production of the system output and is beneficial in reducing the basal expression [Supplementary Table S3; (Qian and Winfree, 2011)]. The slower $k_{s}$ rate caused the time needed to reach the maximum system output to increase from 2,500 s in (Qian and Winfree, 2011) to 20,000 s in the optimal system [Supplementary Figure S11; (Qian and Winfree, 2011)]. This is coupled to a faster $k_{f}$ rate, which ensures faster binding of the input miRNA to the threshold strand, further lowering the basal expression and increasing the threshold accuracy (Supplementary Table S3). To illustrate this, we performed sensitivity analysis that further highlighted this beneficial change in rates compared to (Qian and Winfree, 2011), as both a slower $k_{s}$ rate and a faster $k_{f}$ reduces the fluorescence output at low input dose concentrations (Supplementary Figure S8; (Qian and Winfree, 2011)). Interestingly, decreasing the rate $k_{f}$ decreases the sensitivity of the system to changes in $k_{s}$ , whilst increasing $k_{s}$ decreases the system’s sensitivity to changes in $k_{f}$ across different input miRNA doses (Supplementary Figures S9, S10). The possibility to adjust the rates of the TMSD-F by changing the length and nucleotide composition of the strands suggests that these systems are relatively easy to engineer compared to the protein-based FFL systems.

With these results in mind, the improved threshold accuracy, higher slope and reduced basal expression of the optimised TMSD-F produces the dose-response curve shown in Figure 3C. Compared to our three-node systems, the better engineering possibilities and the absence of oscillations are great advantages for employing the TMSD-F system as the concentration-dependent module in our miRNA diagnostic test.

### 3.3 Optimising toehold systems in the absence of fuel reactions

As the kinetic model of the TMSD-NF system was adapted from TMSD-F, we assumed that the rates of similar reactions could be transferred between the systems (Figure 4A). Therefore, the threshold reaction proceeds again with rate $k_{f}$ , while the TMSD and aptamer binding reaction proceed with rates $k_{s}$ (here named $k_{s1}$ and $k_{s2}$ , respectively).

In simulations with these reaction rates, the TMSD-NF system generates more linear dose-response curves than the TMSD-F system. The TMSD-NF system showed minimal basal expression, but the slope of the dose-response curve was less steep than in the TMSD-F system (Figure 4B, pink line). To test whether the decrease in basal expression was due to removing the fuel reaction, the initial concentration of the fuel component was set to 0 in the TMSD-F system, thereby eliminating the fuel reaction (Supplementary Figure S12). The simulations showed a small increase of basal expression in the absence of the fuel reaction.

To further engineer the TMSD-NF system, the initial concentrations of intermediate output IO (in complex with TH ) and aptamer P were adjusted to observe the trade-off between maximum system output and the slope of the resulting dose-response curve (Supplementary Figures S17, S18). Concentrations of IO and P equal to $\frac{1}{3}K_{\mathrm{expected}}$ produced dose response curves with steep slopes and high levels of fluorescence when miRNA inputs are above $K_{\mathrm{expected}}$ (set to 2 nM in Supplementary Figure S18). Consequently, though, the fluorescent output cannot exceed $\frac{1}{3}K_{\mathrm{expected}}$ , which could be problematic when trying to detect miRNA with low threshold concentrations between off- and on-states.

In Qian and Winfree (2011), the system was designed on the assumption that the threshold reaction should be faster than the TMSD reaction (i.e., $k_{f}>k_{s1}$ ) to produce a dose-response curve that converts the continuous input into a binary output. This principle was transferred to the TMSD-NF system. According to sensitivity analysis of the ODEs, the $k_{f}$ and $k_{s1}$ rates have opposing effects on fluorescent outputs (Supplementary Figure S13). At low input miRNA levels, increasing $k_{f}$ decreased output fluorescence whilst increasing $k_{s1}$ led to increased output. Furthermore, the sensitivities were constant over time, possibly owing to the system reaching steady state almost instantaneously (Supplementary Figure S16). As with the TMSD-F system, we saw that increasing $k_{s1}$ (or decreasing $k_{f}$ ) leads to the system being more robust to changes in $k_{f}$ (or $k_{s1}$ ; Supplementary Figures S14, S15). Therefore, we hypothesised that the behaviour of the system is mostly dependent on the ratio between rates $k_{f}$ and $k_{s1}$ .

To test this observation, a Latin Hypercube sample (Section 2.5) was created of the permissible parameter space to get a better understanding of the relationship between these TMSD-NF parameters. The permissible parameter space is defined by scoring functions s_1_ to s_4_ (Equations 7 - 10). Upon plotting the ratio $\frac{k_{f}}{k_{s1}}$ against the performance score (Equation 11), a clear trend emerged. The larger the ratio, the better the dose-response curve is observed by high score values. From roughly $\frac{k_{f}}{k_{s1}}=10^{3}$ onwards, the TMSD-NF system always manages to produce the intended outcome (Figure 4C). Interestingly, $k_{s2}$ has a defining character when the ratio is small. Even though $k_{f}<k_{s1}$ , a low-binding aptamer (low $k_{s2}$ ) led to a high score. Potentially, the output TMSD strand IO is produced much quicker, but IO must accumulate to larger quantities to overcome the poor aptamer binding imposed by $k_{s2}$ . In this sense, the aptamer P creates the threshold instead of the antisense strand TH . Nonetheless, our results suggest that a threshold reaction should have a $k_{f}$ that is at least 1,000 times faster than $k_{s1}$ . This can be achieved by extending the toehold length of the threshold reaction, thereby increasing $k_{f}$ , or shortening the toehold length of the TMSD reaction described by $k_{s1}$ .

### 3.4 Comparison of the three systems

As many miRNAs can differentiate between healthy people and patients diagnosed with MS, we want to create a system that can respond to a suite of differentially-expressed miRNAs. However, each miRNA will have a different threshold concentration that distinguishes between healthy patients and those with MS. Therefore, the concentration-dependent module requires a modular design that is easily adaptable to new $K_{\mathrm{expected}}$ values.

**FIGURE 5:**
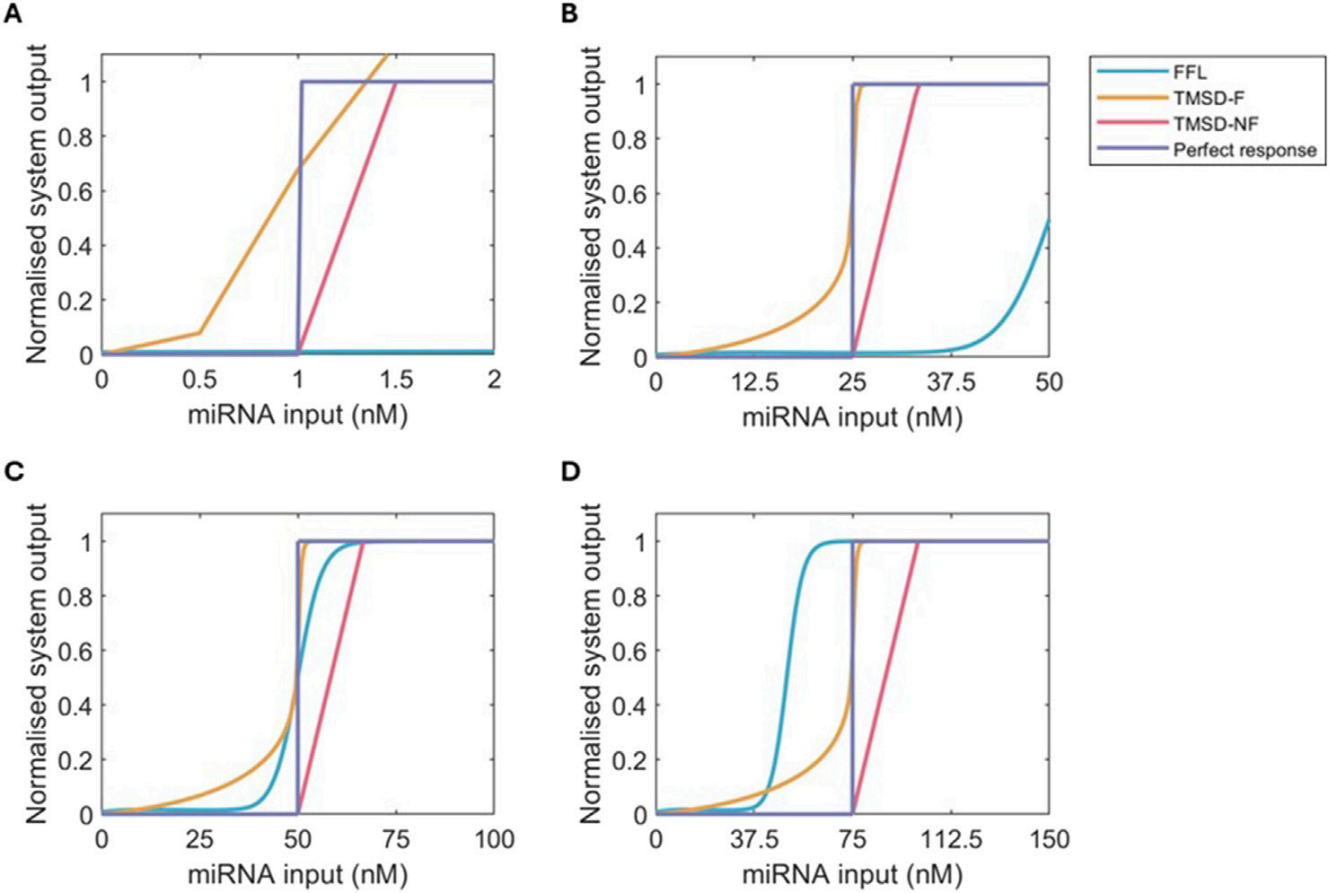
Normalised dose-response curves for FFL (blue), TMSD-F (orange), TMSD-NF (pink) and a perferct binary response (purple) at multiple values of $K_{\mathrm{expected}}$ . The curves were normalised by dividing the fluorescent output for each dose by the maximum system output. **(A)** $K_{\mathrm{expected}}$ = 1 nM. **(B)** $K_{\mathrm{expected}}$ = 25 nM. **(C)** $K_{\mathrm{expected}}$ = 50 nM. **(D)** $K_{\mathrm{expected}}$ = 75 nM.

By changing the input concentrations of the system, where the threshold strand (TH) concentration is equal to $K_{\mathrm{expected}}$ , we can study how adaptive the systems are to new threshold values (Figure 5). The switching behaviour of the TMSD systems (yellow and pink lines) track the value of $K_{\mathrm{expected}}$ used in the simulations, indicating good adaptive behaviour to new input miRNAs necessary for a modular system. Conversely, regardless of input miRNA’s $K_{\mathrm{expected}}$ values, the FFL system will always produce the same dose-response curve, which converts the miRNA input to a binary output at $K_{\mathrm{expected}}$ equal to 50. For a new threshold value, the optimisation of our three-node networks would have to be redone to find the best topology, which is disadvantageous relative to the easy changes in input concentrations that can be made for the TMSD systems.

Focussing back on a $K_{\mathrm{expected}}$ of 50 nM, the FFL system does have a very high threshold accuracy, an acceptable basal expression, and a sufficiently steep slope (Figure 5C). The FFL topology does not oscillate over time resulting in the maximum output of the system, 20 nM, being consistent after the $K_{\mathrm{expected}}$ mark (Supplementary Figure S5). In comparison, the TMSD-NF system has an even lower basal expression and a similar slope, but the maximum output of the system is limited by the $K_{\mathrm{expected}}$ value as discussed above. The incorporation of the fuel reaction in TMSD-F introduces both benefits and limitations compared to the other two systems. The maximum possible fluorescence is the highest of the three systems and can be engineered to be even higher, but this comes at the cost of high basal expression (Figure 5C). Limiting the basal expression further might further slow down the system which would be undesirable depending on the system’s application. The TMSD-F system does accurately detect the threshold, and the slope in the dose-response curve is the steepest out of the three systems.

In the final diagnostic test, our results recommend the use of TMSD systems as the chance of oscillations and the limited scalability of three-node networks, like the FFL system, are undesired. The TMSD-F system has the most potential if further reduction of the basal expression can be achieved. TMSD-F has a high threshold accuracy, the highest slope and the highest maximum system output. The latter point, in particular, is a disadvantage of the TMSD-NF system, where the maximum system output is relatively low. If further optimisation of the TMSD-F system proves difficult, the system would be best employed at low $K_{\mathrm{expected}}$ values as the increase in system output provided by the fuel is most relevant at these input concentrations. At higher $K_{\mathrm{expected}}$ values, the basal expression of the TMSD-F system can increase and cause false positives, so for those $K_{\mathrm{expected}}$ values, the TMSD-NF system might be preferred. Ultimately, we have shown the TMSD systems have the theoretical potential to be simply engineered for application as concentration-dependent modules in a range of miRNA-based detection tools.

## 4 Discussion

In this work, we have utilised a previously published multi-objective optimisation strategy to design biological mechanisms that are capable of converting (continuous) miRNA inputs into binary output signals. As per the last section of the results, the RNA-based TMSD systems outperform the protein-based three node FFL system. These TMSD systems can easily be adapted to other input miRNAs (with different $K_{\mathrm{expected}}$ values), have a high threshold accuracy, and do not cause aberrant behaviour such as oscillations. Crucially, though, our key insights into the functioning of toehold systems are yet to be experimentally validated. The previous success whereby models of toehold systems have been experimentally validated (see the Supplementary Material of Qian and Winfree (2011) for examples) gives us hope that the conclusions we discuss below apply in experimental contexts too. We will highlight here how our modelling framework can be extended to account for more biological detail, which experiments would be needed to provide more information to our model, and how our results can be translated into experimental insights by future iGEM teams and researchers.

### 4.1 TMSD engineering

The fuel reaction in particular is an interesting target for optimising the TMSD-F system further. Our results showed that this reaction in the TMSD-F system results in a trade-off between a high fold-change in the system output versus lower basal expression (Figure 3B). The optimisation results showed that the basal expression can be decreased by lowering the $k_{s}$ rate describing the fuel, TMSD, and reporter reaction (Figure 3A, reactions II, III and IV, respectively). Biologically, this could be achieved by shortening the toehold length which initiates the reaction (Zhang and Winfree, 2009; Machinek et al., 2014). In their Supplementary Material, Qian and Winfree (2011) proved — with a mix of modelling and experimental work — that a smaller toehold length decreased basal expression of output reporters (Qian and Winfree, 2011). The fuel concentration that best balances increased system output with basal expression is an interesting problem for future design strategies. However, the fact that lower basal expressions might result in lower threshold accuracies should not be forgotten.

A critical limitation of the current TMSD-F and TMSD-NF models is the systems’ reliance on domain binding (i.e., toehold to toehold) rather than sequence-specific binding. While, for example, the length of the toehold is essential for the speed of the TMSD reaction rates, the sequence itself also plays a role (Zhang and Winfree, 2009; Berleant et al., 2018). This indicates that the $k_{s}$ rates might differ for the TMSD, fuel and reporter reactions as their sequences are not completely identical, which is currently an assumption we are making within our models. To account for this, the mathematical models could be further refined by including three separate $k_{s}$ rates. Moreover, modelling and optimising three separate $k_{s}$ rates could aid in accelerating the speed of TMSD-F systems. For example, a faster fuel reaction rate might speed up the re-use of input miRNA, while TMSD and reporter reaction rates are kept lower to reduce the basal expression. Additionally, replacing the universal toehold with reaction-specific toeholds could help prevent unintended side reactions, in which incompatible strands temporarily bind at the universal toehold without completing the reaction. Eliminating these non-productive interactions would likely speed up the reaction dynamics and result in faster fluorescent production. Furthermore, slight alterations in the secondary structure of the strands in the TMSD system could decrease their free energies and speed up the reaction (Jung et al., 2022). To experimentally verify the role of separate $k_{s}$ rates, future experiments could be directed to tracking the individual concentrations of the compounds over time or applying TMSD rate prediction algorithms to find more specific $k_{s}$ rates (Akay et al., 2024; Berleant et al., 2018).

Including sequence-specificity in the model becomes even more evident when considering the application of the TMSD system in diagnostic tests. Ultimately, our designed system would be used in applications to detect multiple miRNAs simultaneously, meaning that multiple TMSD systems will need to work in parallel. Here, sequence specificity becomes crucial, as the wrong miRNA should not trigger a TMSD reaction and produce false positives or negatives. In this work, we assumed that parallel detection is possible, allowing us to model one TMSD system that can be repurposed for all miRNA that we wish to detect. This assumption could potentially be violated on sequence level, which could, for example, lead to a decoy miRNA with a slight mismatch to bind to the threshold strand of the target miRNA. This could cause false positives where the concentration of the target miRNA did not pass the threshold but, together with the decoy miRNA, the threshold is surpassed. This signifies the need for well-designed toeholds that are highly specific for one miRNA only. Fortunately, when TMSD was used as an amplification module, it was specific to single nucleotide mismatches (Zhang et al., 2020). Other work underlines the importance of sequence specificity, but current models of this mismatch effect are dependent on specific toehold lengths and the position of the mismatching nucleotide (Machinek et al., 2014). The incorporation of precise sequence design into the model could clarify whether the reaction is specific enough to detect particular miRNAs.

To tackle these issues of sequence specificity and decoy miRNAs for our designed systems, we propose three extensions to our work for practitioners and future research through the use of Figure 4C. In the first instance, our modelling framework could be extended to incorporate sequence specificity by making use of the previously developed KinDa tool. This tool compares the functioning of TMSD systems at the domain and sequence level with stochastic modelling (Berleant et al., 2018). This way, the behaviour of nucleotide-specific sequence designs can be checked on the domain level. Furthermore, KinDa can predict the kinetic rates of both the toehold binding and the branch migration reactions that form the TMSD system (e.g. Figure 4A). Modifications to the secondary structure can also be tested with KinDa. Therefore, before implementing TMSD-NF in the lab, proposed sequence designs could be evaluated with KinDa, ruling out any disturbing side reactions, and the predicted kinetic rates could be cross-referenced with the $k_{f}/k_{s1}$ ratio found through Latin hypercube sampling (Figure 4C). Those sequences that utilise $k_{f}/k_{s1}$ ratios with high scoring outputs could then be considered as acceptable TMSD mechanisms for our tested biomarker miRNA.

Alternatively, extra experimental data could be obtained to further evaluate the performance of TMSD systems. For example, in the first instance, practitioners could evaluate the performance of the TMSD system with varying amounts of initial aptamer concentration or testing aptamers of different binding strength. As we observe in Figure 4C, when the parameter $k_{s2}$ (related to concentration of aptamer) is varied then at low $k_{f}/k_{s1}$ ratios we observe varying TMSD performance. Consequently, if TMSD performance is shown to depend on aptamer concentration or sequence then this correlates with a low $k_{f}/k_{s1}$ ratio suggesting there is an issue within the system (e.g. decoy miRNAs or nucleotide mismatches could be present in the sample perturbing the TMSD). Second, the robustness of a TMSD system’s response to a particular miRNA could be evaluated in conjunction to dose-response curves obtained when using decoy miRNA or miRNA with mismatching nucleotides as inputs. In the event that significant overlap of dose-response curves is observed between these conditions, then this is suggestive of the TMSD system being insensitive to changes in nucleotide sequence since mismatching miRNA inputs can trigger the TMSD system as well as perfectly matching miRNA inputs. In both instances — either when TMSD systems are sensitive to aptamer alterations or TMSD performance significantly overlaps between perfect and mismatching miRNA inputs — then we would encourage testing other TMSD designs for other potential biomarkers to find robust detection mechanisms.

### 4.2 Improving optimisation for better design of FFL threshold mechanisms

A major issue in our three-node network designs is the formation of topologies that cause oscillations over time in the fluorescent output. For a correctly working threshold mechanism, the system should reach steady state within a reasonable time period. Otherwise, the output signal is inconsistent, and accurate measurements of the miRNA concentrations are difficult. Although the topologies of the networks causing oscillations and the networks resulting in smooth dose-response curves do not entirely overlap, they share heavy negative regulation on node A (Figure 4A; Supplementary Figure S6). In previous studies, this type of negative feedback has been associated with networks that provide robustness to noise, as well as oscillations (Kholodenko, 2006; Holehouse et al., 2020; Tyson and Novák, 2010). The former is beneficial for the functioning of the threshold mechanism if the input doses below $K_{\mathrm{expected}}$ are considered noise. Noise suppression was observed in the time responses of well-functioning threshold mechanisms, where an initial peak was followed by downregulation to a low fluorescent output level. The motif of node A activating node B , which in return inhibits A , has been specifically linked to noise suppression and is an important part of the best-performing topologies (Tyson and Novák, 2010). It might be interesting to explore if even stronger negative regulation on node A reduces the time it takes for the system output C to reach steady state (Figure 2D). This system speed up would be beneficial since, with our current results, there is the potential that the initial peak of output C might accidentally trigger the detection module, which comes after the concentration-dependent threshold module.

However, strong negative feedback is also associated with oscillating networks (Kholodenko, 2006; Tyson and Novák, 2010). The specific motif responsible for the oscillations found in our optimisation strategy are known as Goodwin oscillators, and the network’s dynamics have been extensively studied as the network motif has been found in circadian clocks (Goodwin, 1965; Ullner et al., 2009; Baum et al., 2016; Gonze et al., 2005). Furthermore, negative autoregulation on node A is associated with robust oscillatory behaviour (Woods et al., 2016). In the optimisation of our three-node networks, this negative autoregulation was more often observed in the topologies with smooth dose-response curves than in the topologies resulting in oscillations. It has been proposed that the properties of negative autoregulation depend on the other parameters in the system, which might explain negative autoregulation being a part of both oscillating and threshold mechanism FFL systems (Marquez-Lago and Stelling, 2010). This illustrates that further careful and robust design of the FFL system is necessary before it is tested in the lab. If the behaviour of the dose-response curve is highly dependent on the exact strengths and node connections found with the optimisation, it might not work as well as it should in the final test.

Therefore, adding a constraint to the optimisation strategy that prevents any solutions with oscillations would be necessary. This could be achieved by adding careful constraints to which reactions within a network are allowed, and is required since our current constraints are insufficient to achieve this currently. Alternatively, the method of Otero-Muras and Banga (2016) used to find three-node topologies capable of oscillations could be adapted to filter out oscillations in the FFL optimisation through alterations of their scoring functions. Their oscillation constraint was based on the autocorrelation function, which determines how well the peaks of oscillations align over time. By assessing the behaviour of these constrained designs (e.g., lower basal expression), we could potentially obtain general design principles for robust miRNA detection tools. With these design principles to hand, other system properties, such as robustness or the effects of stochastic behaviour, could further distinguish the solutions (Woods et al., 2016).

If we take a step back and evaluate the multi-objective optimisation framework as a whole, we have observed trade-offs between different objectives through the visualisation of our search spaces in Figures 2B, 3B. However, decisions cannot be based on this information alone. Careful examination of the dose-response curves and time-responses was necessary to filter out undesired behaviour, like oscillations produced by three-node networks, and determine the influence of a smaller $k_{s}$ on the time required to reach the steady state in the TMSD-F optimisation. With additional constraints, these filtering steps could be included in future design strategies based on the approach used here.

## 5 Conclusion

In summary, this study modelled and explored three biological mechanisms in their ability to convert continuous miRNA input into a binary output above a specific threshold. All the system designs studied here showed potential for future use in sensor- or diagnostic tests. However, the RNA-based TMSD systems are easier to engineer, more stable, and more adaptable to new input miRNAs than protein-based networks such as the FFL system. The TMSD-F system would outcompete the TMSD-NF system at higher threshold values if the basal expression produced by our TMSD-F design could be further reduced.

In the future, the miRADAR project of the WUR iGEM 2024 team envisions the incorporation of this concentration-dependent module into cell-free miRNA diagnostic tests (iGEM, 2024). The threshold mechanism allows clear separation of input miRNA concentrations into a binary output to distinguish miRNA concentrations of healthy people from those with MS, which is a feature lacking in current tests. The adaptability of the TMSD system to new sequences and thresholds enables the test to be modified for other diseases besides multiple sclerosis, further highlighting the importance of continued research into these concentration-dependent modules.

## Data Availability

The datasets presented in this study can be found in online repositories. The names of the repository/repositories and accession number(s) can be found below: https://git.wur.nl/ssb/publications/designing-mirna-detection-networks.
