# Supplementary material for: A model-based design strategy to engineer miRNA-regulated detection systems
Source: Front Syst Biol. 2025 Aug 14;5:1601854. doi: 10.3389/fsysb.2025.1601854 (PMC12390972; doi:10.3389/fsysb.2025.1601854)
Supplement: Supplementary file 1 [file DataSheet1.pdf]

Supplementary Methods

Contents

S1 Computational methods **2**
S1.1 Determining the optimal number of doses to simulate in the multi-objective optimisation 2
S1.2 Evaluation of scoring metrics 4
S1.3 ODEs for the FFL system 7
S1.4 ODEs for TMSD-F 8
S1.5 ODEs for TMSD-NF 12
S1.6 Sensitivity analysis 12

S2 Supplementary results **14**
S2.1 FFL 14
S2.2 TMSD-F 17
S2.3 TMSD-NF 22
S2.3.1 Determining the best ratio of initial concentrations 25

S1 Computational methods

S1.1 Determining the optimal number of doses to simulate in the multi-objective optimisation

The ODEs had to be simulated for multiple input doses to generate a dose-response curve. Simulating a large number of doses would slow down the optimisation drastically, so a minimal amount of doses, which still accurately represent the curve, had to be determined. For this, synthetic data sets were created by simulating the Hill function, which mathematically describes the dose-response curve as follows

$$O = I + O_{max} \frac{D^n}{K_{Hill}^n + D^n}, \tag{1}$$

where K_{Hill} is the concentration of input miRNA D resulting in half of the maximum system output O [1]. The slope of the curve is denoted by n and the intercept I is the system output O at $D = 0$.

In each data set, the number of doses close to $K_{expected}$, referred to as K_{points}, was varied, as this region (R2 in Figure S1A) is the most variable. The K_{points} are equally distributed in the R2 region, whose size is defined as $K_{expected} \pm \frac{K_{expected}}{\alpha}$. Multiple values for α were initially explored but $\alpha = 3$ was found to give the best results. The doses before and after the switch area R2 are defined as five evenly spaced points: for R1, between $D = 0.01$ and the first K_{point}, and for R3, between the last K_{point} and $D = 4 \times K_{expected}$ (Figure S1A). The output of the system O is extrapolated from these doses with the Hill function. Noise was added to the outputs to represent the wet lab environment and to infer a more robust K_{points} value with

$$O_{noise} = O + \rho O_{max} \epsilon, \tag{2}$$

where the perturbation level ρ was set to 0.3 and $\epsilon \sim N(0, 1)$. The results were verified for $\rho = 0.1$ and $\rho = 0.5$. In total, ten noisy datasets were made. The Hill function parameters K and n were reverse calculated from each of the ten noisy datasets, after which outliers were removed before the values were averaged into a mean value. The mean K and n were compared to their expected values and plotted in a bar graph to evaluate the best number of K_{points} (Figure S1B). The number of K_{points} did not cause significant differences (Figure S1B left). In contrast, the mean slope accuracy, especially for higher slope values, depends on the number of K_{points}. The more points, the better, although the effect seems to saturate from $K_{points} = 10$ onwards (Pink bars in Figure S1B right). Therefore, K_{points} was set to 10 in both the FFL and TMSD-F optimisations to ensure good accuracies while preserving computational viability.

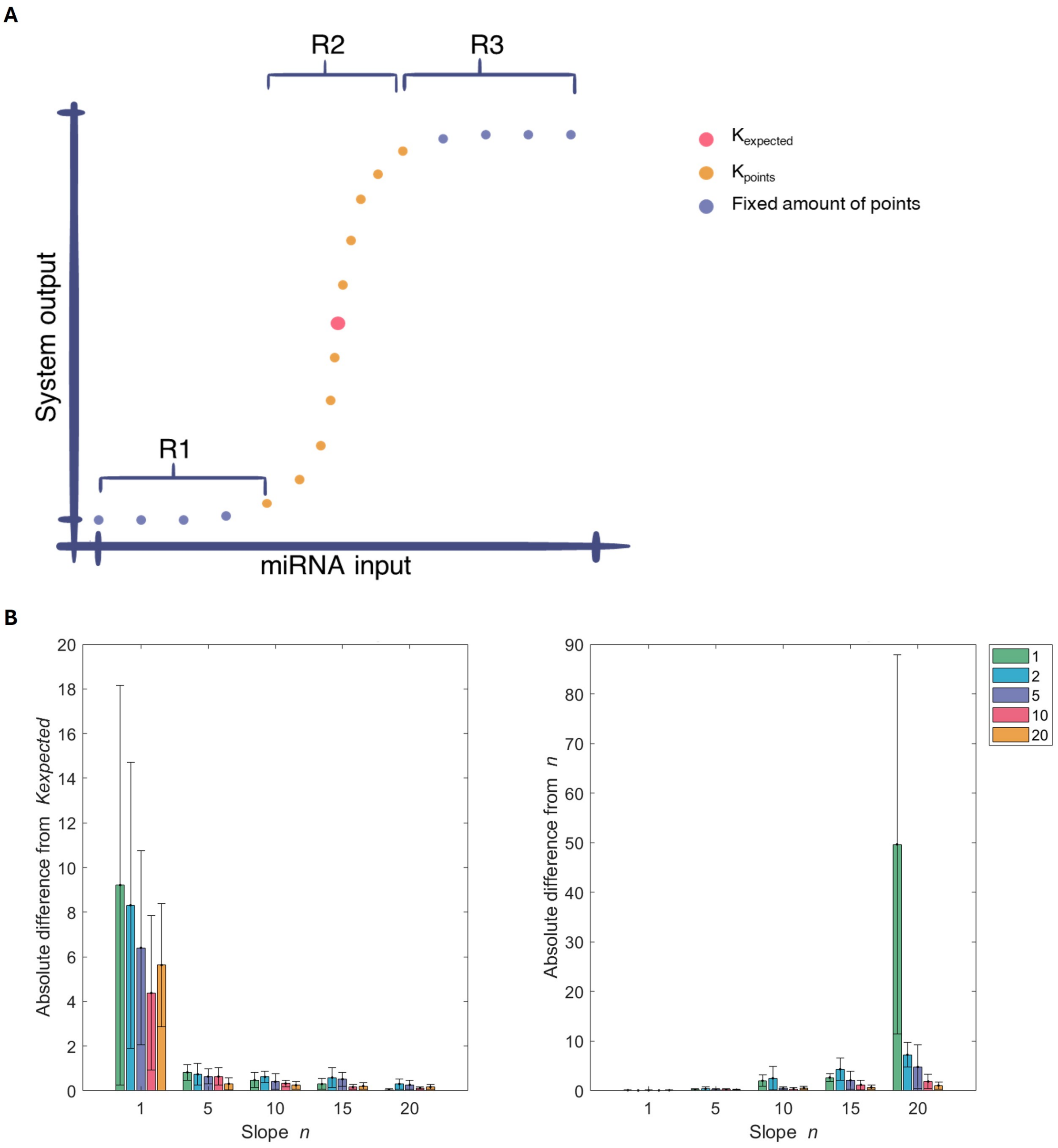

Figure S1: Finding the optimal number of points to simulate in a dose-resopnse curve. A) The rationale for finding the minimal amount of doses needed to accurately simulate a dose-response curve. R1 consists of five equally spaced points between an input miRNA concentration of 0.01 and the first K_{point}. R3 consists of five equally spaced points between an input miRNA concentration of the last K_{point} and $4 \times K_{expected}$. The size of R2 is defined as $K_{expected} \pm \frac{K_{expected}}{3}$. The number of K_{points} was varied and evaluated. B) The mean threshold accuracy (left) and mean slope accuracy (right) for different values of slope n (x-axis) and amount of K_{points} (coloured for). The simulations were performed with $K_{expected} = 50$. For each slope value and K_{point}, 10 replicate datasets were generated with a noise perturbation value of $\rho = 0.3$.

S1.2 Evaluation of scoring metrics

In the main text, we present scoring metrics for our optimisation strategy (equations (2) to (11)). The choices for scoring metrics contain assumptions that, here, we will evaluate and justify further.

In the first instance, above in Section S1.1 we discuss how we select the number of doses to simulate in region R2 and choose five evenly-spaced points in region R1 to simulate (Figure S1A). In the main text, we also explain that in f_3 and s_2, we evaluate a system's ability to reduce output expression at low doses by summing output expression across these doses in R1. Alternative metrics that provide the same information are possible, but we consider this relative to the number of doses that are evaluated. In Figure S2 (left) we show the sum of output levels in region R1 for our optimal FFL system increases, as expected, linearly when more doses are added to the evaluation. Comparatively, an alternative metric is the mean of the output levels (or, output level per evaluated dose) across this region. This alternative metric shows that the output expression per dose does not greatly change as more doses are evaluated in region R1 to optimise FFL systems. Consequently, we argue that evaluating more doses in region R1 would not greatly alter our conclusions: if a system has low total expression levels in region R1 then the output level per evaluated dose would also remain low (as desired). At the same time, increasing the number of evaluated doses increases computational time for our FFL optimisation strategy (Figure S2 (right)). This suggests that evaluating more doses in region R1 or using alternative metrics would not change our conclusions in relation to whether or not our optimal design suppress output expression in region R1 but could negatively impact the computational time needed to evaluate different designs.

Another scoring metric choice that we have made was to alter f_4 used in the FFL and TMSD-F optimisation to metric s_3 for TMSD-NF. This change was required for practical reasons since the TMSD-NF system shows steep switching behaviour at doses, D, beyond $K_{expected}$ (see Figure 4B of the main text and E below for examples). This means that fitting Hill functions for defined and fixed $K_{expected}$ values (as in equation (1) above) is not possible. As a consquence, rather than estimating n from fitted curves (as in metric f_4) we evaluated the behaviour of our dose-response curves beyond $K_{expected}$ to judge the steepness of the switching behaviour (as in metric s_3). Here, we show how these two metrics compare to one another.

In Supplementary Figure , we show how simulations of equation (1) above compare for different values of n. As expected, as n increases, the dose-response curve becomes steeper. To evaluate the metric s_3, we obtain

$$\frac{dO}{dD} = O_{max} D^n K_{Hill}^n \frac{\ln D + \ln K_{Hill}}{\left(D^n + K_{Hill}^n\right)^2}, \tag{3}$$

$$\frac{d}{dn}\left(\frac{dO}{dD}\right) = O_{max} D^{n-1} K_{Hill}^n \frac{\left(D^n + K_{Hill}^n\right)\left(1 + n\ln D + n\ln K_{Hill}\right) - 2n\left(D^n \ln D + K_{Hill}^n \ln K_{Hill}\right)}{\left(K_{Hill}^n + D^n\right)^3}, \tag{4}$$

and show that $\frac{dO}{dD}$ increasingly peaks around $K_{expected}$ when n (the steepness of the dose-response) increases (Figure S3B). From these curves we can now show that metric s_3 broadly increases with

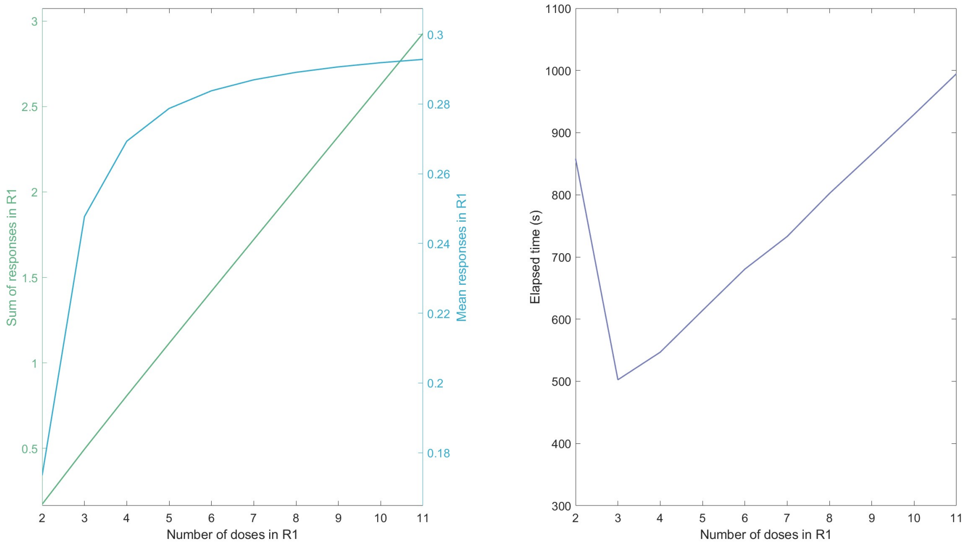

Figure S2: Evaluating behaviour of optimisation strategy when considering different numbers of doses in f_3 from the main text. Left) The sum (green line) and mean of responses (blue line) in region R1 when different numbers of doses from R1 are simulated using our optimal FFL system. Right) Computational time required to perform 10000 simulations (equivalent to one optimisation round in the optimisation algorithm) when varying the number of doses evaluated in region R1 of our optimal FFL network.

n until we reach $n \approx 6$ (Figure S3C) and that increasing n further does not yield great changes in this score (Figure S3D). Whilst this suggests that we may not be able to accurately backcalculate a value for n from systems with comparably high s_3 scores, we conclude from this that increasing s_3 does correlate with an increased steepness of the dose response curve, n or f_4. As such, if a system has a steep switching behaviour, or high n/f_4, then the system will also have a high score s_3. We highlight this with examples of our optimal FFL and TMSD systems (Figure S3E & F).

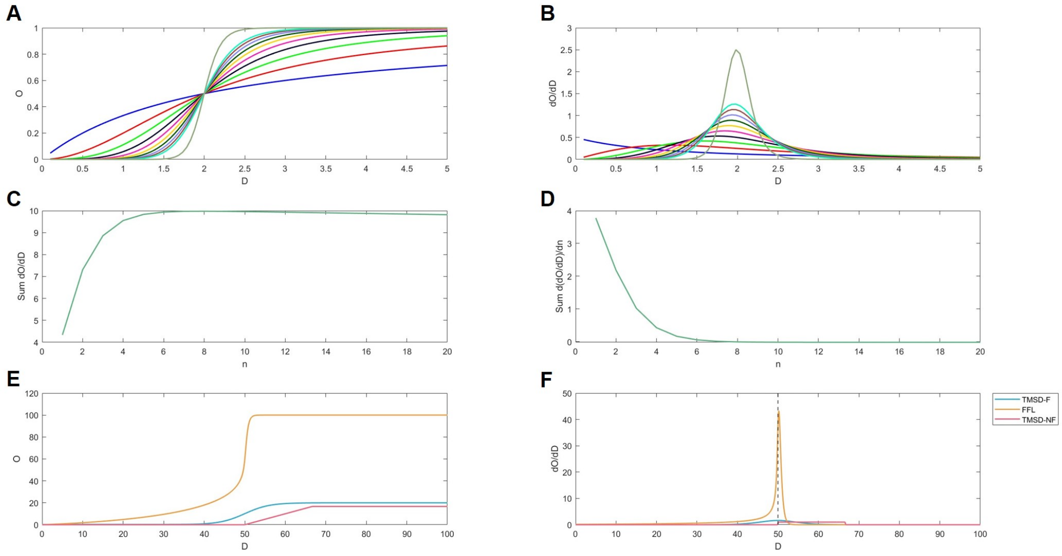

Figure S3: Comparison of metric behaviour for f_4 and s_3 from the main text. (A) Simulations of supplementary equation (1) with $I = 0$, $O_{max} = 1$, and $K_{Hill} = 2$ for different values of n across a dose-range from $D = 0$ to $D = 5$. (B) Simulations of dO/dD (supplementary equation (3)) for different values of n. (C) The sum of dO/dD from $D = K_{expected} = 2$ to $D = 5$ as per scoring function s_3 versus parameter n. (D) The sum of $d(dO/dD)dn$ from $D = K_{expected} = 2$ to $D = 5$ versus parameter n. (E) Optimal dose-response curves obtained from the FFL (orange line), TMSD-F (blue line) and TMSD-NF (red line) systems. The normalised versions of these dose-response curves are presented in Figure 4B of the main text. (F) Plots of dO/dD for our optimal systems against dose D. Line colours are as in panel (E).

S1.3 ODEs for the FFL system

The following ODEs were taken from [5] and describe the three nodes A, B, and C in the FFL system respectively

$$\frac{dA}{dt} = \frac{1}{1 + exp(a - b(D + \omega_{AA}A + \omega_{BA}B + \omega_{CA}C))} - \delta A \tag{5}$$

$$\frac{dB}{dt} = \frac{1}{1 + exp(a - b(\omega_{AB}A + \omega_{BB}B + \omega_{CB}C))} - \delta B \tag{6}$$

$$\frac{dC}{dt} = \frac{1}{1 + exp(a - b(\omega_{AC}A + \omega_{BC}B + \omega_{CC}C))} - \delta C, \tag{7}$$

where D is the input miRNA concentration, a and b are constants from the promoter regulation function, ω the connection between two nodes ($w_{ij} = x_{ij}y_{ij}$) and δ the protein degradation rate constant. The mRNA dynamics of the proteins A, B and C are assumed to be on a different timescale such that the network can be simplified into three protein ODEs. The constants a, b and δ facilitate this. All values and optimisation boundaries for the parameters are in Table S1. The system was simulated for 1500 seconds to ensure steady state behaviour. Initial concentrations of nodes A, B and C were set to 0.1 nM [5].

Table S1: Parameters of the ODEs for the FFL system with their respective values or boundaries.

**Parameter**	**Meaning**	**Boundary in optimisation**	**Unit**	**Source**
x_{ij}	Strength of the regulation	$[10^{-3}, 20]$ $\forall$ $i, j \in \{A, B, C\}$	Dimensionless	[5]
y_{ij}	Type of regulation; -1: inhibition, 1: activation, 0: no action	$1, -1$, or 0 $\forall$ $i, j \in \{A, B, C\}$	Dimensionless	[5]
ω_{ij}	$x_{ij}y_{ij}$, the connection between two nodes	-	Dimensionless	[5]
a	Steepness of the threshold value of the regulation function	Fixed to 5	Dimensionless	[5]
b	Location of the threshold value of the regulation function	Fixed to 1	$\frac{1}{nM}$	[5]
δ	Protein degradation	Fixed to 0.05	s^{-1}	[5]

S1.4 ODEs for TMSD-F

All values and optimisation boundaries for the parameters are in Table S2. The following reactions describe the full TMSD-F system with first the threshold, TMSD, fuel and reporter reaction

$$D + TH \xrightarrow{k_f} W \tag{8}$$

$$D + C1 \underset{k_s}{\overset{k_s}{\rightleftharpoons}} C2 + IO \tag{9}$$

$$C2 + F \underset{k_s}{\overset{k_s}{\rightleftharpoons}} C3 + D \tag{10}$$

$$IO + R \xrightarrow{k_s} O, \tag{11}$$

where D is the input miRNA, TH is the threshold complex, W is a waste product of that reaction, C1 is the TMSD complex with intermediate output strand IO on it at first. After the displacement of IO from C1 by D, C2 is formed. C3 is the complex formed after fuel F displaces D from C2. Intermediate output strand IO from equation (9) reacts with the reporter complex R to create fluorescent output O.

The forward and reverse reactions in equations (9-11) are initiated by the same universal toehold and thus proceed with the same reaction rate k_s. Equations (9) and (10) are reversible, as the product complexes (C2 and C3, respectively) have a free toehold, allowing the other product strands (IO and D, respectively) to initiate the reverse reaction. There is no free toehold left in the products of equations (8) and (11), making them irreversible.

The universal toehold reactions can be described as

$$IO + C1 \underset{k_{rf}}{\overset{k_f}{\rightleftharpoons}} C4 \tag{12}$$

$$F + C1 \underset{k_{rf}}{\overset{k_f}{\rightleftharpoons}} C5 \tag{13}$$

$$IO + C3 \underset{k_{rf}}{\overset{k_f}{\rightleftharpoons}} C6 \tag{14}$$

$$F + C3 \underset{k_{rf}}{\overset{k_f}{\rightleftharpoons}} C7 \tag{15}$$

$$C2 + D \underset{k_{rf}}{\overset{k_f}{\rightleftharpoons}} C8 \tag{16}$$

$$IO + TH \underset{k_{rs}}{\overset{k_f}{\rightleftharpoons}} C9 \tag{17}$$

$$F + TH \underset{k_{rs}}{\overset{k_f}{\rightleftharpoons}} C10 \tag{18}$$

$$D + R \underset{k_{rf}}{\overset{k_f}{\rightleftharpoons}} C11 \tag{19}$$

$$F + R \underset{k_{rf}}{\overset{k_f}{\rightleftharpoons}} C12, \tag{20}$$

where C4 to C12 are complexes formed by the binding of the universal toeholds of strands and complexes that cannot complete the TMSD reaction. The rate k_{rs} is a slower reverse rate than k_{rf} because of the longer toehold in the threshold strand. Forward reactions of the universal toehold side reactions proceed with rate k_f as they both reach the same maximum hybridisation rates as k_f in equation (8). Moreover, a leakiness reaction is included in the kinetic model. This reaction describes the displacement of the intermediate output IO by fuel F without binding to the toehold, which is modelled as

$$C1 + F \underset{k_l}{\overset{k_l}{\rightleftharpoons}} C3 + IO \tag{21}$$

The initial conditions were calculated from the $K_{expected}$ with $TH(0) = K_{expected}$, $C_1(0) = 2K_{expected}$, $R(0) = 3K_{expected}$ and $F(0) = 4K_{expected}$. $K_{expected}$ is a user-input parameter in the optimisation algorithm that is equal to the expected value at which the threshold should be.

The ranges used for different parameters are presented in Table S2. Note that to reproduce the simulations in [6], units of $\text{nM}^{-1}\text{s}^{-1}$ were required.

The reactions can be translated into the following ODEs:

$$\frac{dD}{dt} = -k_sDC1 + k_sC2IO - k_sDC3 + k_sC2F - k_fDTH - k_fC2D + k_{rf}C8 - k_fDR + k_{rf}C11 \tag{22}$$

$$\frac{dC1}{dt} = -k_sDC1 + k_sC2IO - k_fIOC1 + k_{rf}C4 - k_fFC1 + k_{rf}C4 - k_lC1F + k_lC3IO \tag{23}$$

$$\frac{dC2}{dt} = k_sDC1 - k_sC2IO + k_sDC3 - k_sC2F - k_fC2D + k_{rf}C8 \tag{24}$$

$$\begin{aligned}\frac{dIO}{dt} &= k_sDC1 - k_sC2IO - k_sIOR - k_fIOC1 + k_{rf}C4 - k_fIOC3 \\ &+ k_{rf}C6 - k_fIOTH + k_{rs}C9 + k_lC1F - k_lC3IO\end{aligned} \tag{25}$$

$$\frac{dC3}{dt} = -k_sDC3 + k_sC2F - k_fIOC3 + k_{rf}C6 - k_fFC3 + k_{rf}C7 + k_lC1F - k_lC3IO \tag{26}$$

$$\begin{aligned}\frac{dF}{dt} &= k_sDC3 - k_sC2F - k_fFC1 + k_{rf}C5 - k_fFC3 + k_{rf}C7 - k_fFTH + k_{rs}C10 \\ &- k_fFR + k_{rf}C12 - k_lC1F + k_lC3IO\end{aligned} \tag{27}$$

$$\frac{dTH}{dt} = -k_fDTH - k_fIOTH + k_{rs}C9 - k_fFTH + k_{rs}C10 \tag{28}$$

$$\frac{dW}{dt} = k_f DTH \tag{29}$$

$$\frac{dR}{dt} = -k_s IOR - k_f DR + k_{rf} C11 - k_f FR + k_{rf} C12 \tag{30}$$

$$\frac{dO}{dt} = k_s IOR \tag{31}$$

$$\frac{dC4}{dt} = k_f IOC1 - k_{rf} C4 \tag{32}$$

$$\frac{dC5}{dt} = k_f FC1 - k_{rf} C5 \tag{33}$$

$$\frac{dC6}{dt} = k_f IOC3 - k_{rf} C6 \tag{34}$$

$$\frac{dC7}{dt} = k_f FC3 - k_{rf} C7 \tag{35}$$

$$\frac{dC8}{dt} = k_f C2D - k_{rf} C8 \tag{36}$$

$$\frac{dC9}{dt} = k_f IOTH - k_{rs} C9 \tag{37}$$

$$\frac{dC10}{dt} = k_f FTH - k_{rs} C10 \tag{38}$$

$$\frac{dC11}{dt} = k_f DR - k_{rf} C11 \tag{39}$$

$$\frac{dC12}{dt} = k_f FR - k_{rf} C12 \tag{40}$$

Table S2: Parameters of the ODEs for the TMSD-F system with their respective values or boundaries.

**Parameter**	**Meaning**	**Boundary in optimisation**	**Unit**	**Source**
k_f	Fast kinetic rate; for the threshold reaction and universal toehold binding reactions	$[10^4, 0.5 \times 10^8]$	$nM^{-1}s^{-1}$	[3, 6, 7]
k_s	Slow kinetic rate; for the TMSD reaction and the reporter reaction	$[10^2, 10^7]$	$nM^{-1}s^{-1}$	[6], [7, 3]
k_{rf}	Fast reverse kinetic rate of the universal toehold binding reaction	$[10^{-2}, 10^2]$	s^{-1}	[6], [7, 3]
k_{rs}	Slow reverse kinetic rate of the universal toehold binding reaction	$[10^{-2}, 50]$	s^{-1}	[6], [7, 3]
k_l	Leakiness	$[10^{-3}, 50]$	$nM^{-1}s^{-1}$	[6], [7, 3]

Units were rescaled to $nM^{-1}s^{-1}$ from $M^{-1}s^{-1}$ used in [6]. See comments in text.

S1.5 ODEs for TMSD-NF

The TMSD-NF equations and kinetic rates for TMSD-NF were adapted from TMSD-F to

$$D + TH \xrightarrow{k_f} D\text{-}TH \tag{41}$$

$$D + TH\text{-}IO \underset{k_{s1}}{\overset{k_{s1}}{\rightleftharpoons}} IO + D\text{-}TH \tag{42}$$

$$IO + P \xrightarrow{k_{s2}} O, \tag{43}$$

where D is the input miRNA, TH is the antisense strand to create the threshold and TH-IO is the TMSD complex that will release strand IO, which can consequently bind to aptamer P to create fluorescent output O. In other work, these reactions were modelled in NUPACK, which concluded that no side reactions occurred [4]. Furthermore, it is assumed that TH and IO will dimerise before IO can bind to P and before D is added to the system. The system was simulated for 10 seconds, within which it reached steady state behaviour (Supplementary Materials Section 2.3). Initial concentrations were set as $TH(0) = K_{expected}$ and $TH\text{-}IO(0) = P(0) = 0.5nM$.

The reactions can be translated into the following ODEs:

$$\frac{dD}{dt} = -k_f DTH - k_{s1} DTH\text{-}IO + k_{s1} D\text{-}THIO; \tag{44}$$

$$\frac{dTH}{dt} = -k_f DTH; \tag{45}$$

$$\frac{dD\text{-}TH}{dt} = k_f DTH + k_{s1} DTH\text{-}IO - k_{s1} D\text{-}THIO; \tag{46}$$

$$\frac{dTH\text{-}IO}{dt} = -k_{s1} DTH\text{-}IO + k_{s1} D\text{-}THIO; \tag{47}$$

$$\frac{dIO}{dt} = k_{s1} DTH\text{-}IO - k_{s2} IOP - k_{s1} D\text{-}THIO; \tag{48}$$

$$\frac{dP}{dt} = -k_{s2} IOP; \tag{49}$$

$$\frac{dO}{dt} = k_{s2} IOP; \tag{50}$$

S1.6 Sensitivity analysis

A sensitivity analysis was performed on the best-performing TMSD-F systems with the MATLAB odeSensitivity function under standard parameters. After fixing the parameter values, this function computes the Jacobian matrix and subsequently the sensitivities. The sensitivities U_{ij} were normalised for their parameter value p and component concentration y to show the percentage change under small parameter perturbations, resulting in:

$$U_{ij} = \frac{\partial y_i}{\partial p_j} \frac{p_j}{y_i}, \tag{51}$$

with $i = 1, 2, ..., 5$ and $j = 1, 2, ..., 19$ for TMSD-F and $i = 1, 2, 3$ and $j = 1, 2, ..., 7$ for TMSD-NF [2].

The sensitivities of the optimised TMSD-F and TMSD-NF system to small parameter perturbations were analysed under three conditions: one where the dose is below, one above, and one at $K_{expected}$.

S2 Supplementary results

S2.1 FFL

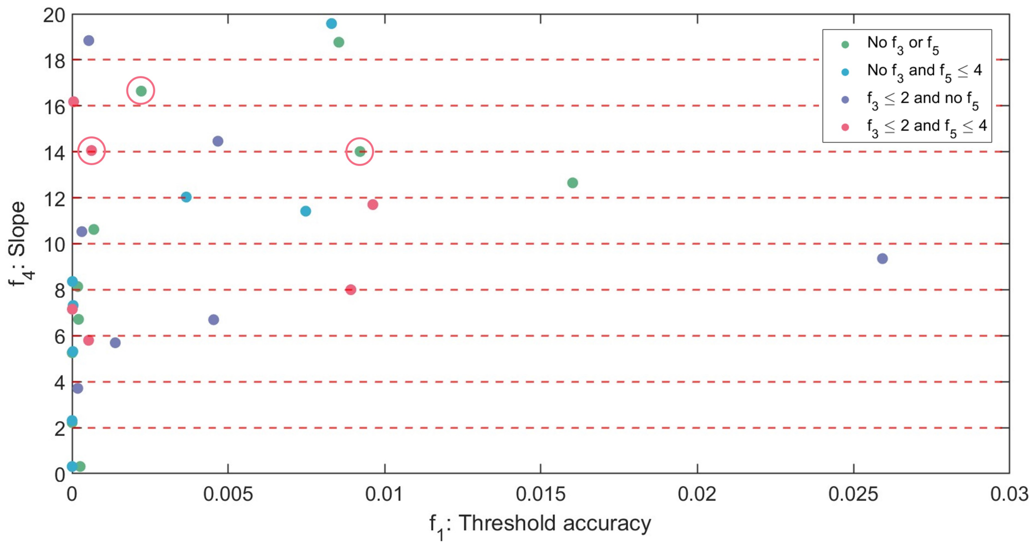

Figure S4: Pareto plot for the FFL optimisation displaying the threshold accuracy (f_1; absolute difference from $K_{expected}$ of 50 nM) and slope (f_4; n from Hill function). The solutions are coloured according to their constraints on f_3 (basal expression) and f_5 (number of node connections). Red dashed lines show slope optimisation boundaries. Encircled solutions are considered the best solutions from the whole optimisation. $K_{expected}$ was set to 50 nM.

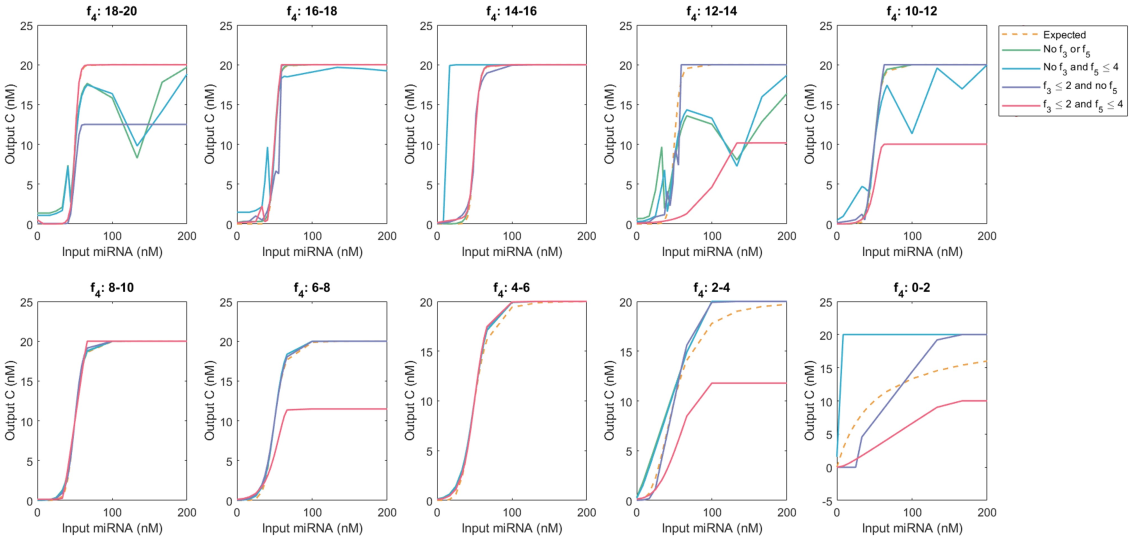

Figure S5: Comparative dose-response curves from the FFL optimisation. Each subplot shows a slope (f_2) constraint interval. Expected curves are computed with the Hill function (intercept = 0.01, maximum output = 100 and slope = average of the interval) and plotted in pink. The other solutions are coloured according to their additional constraints on f_3 and f_5. $K_{expected} = 50$ nM.

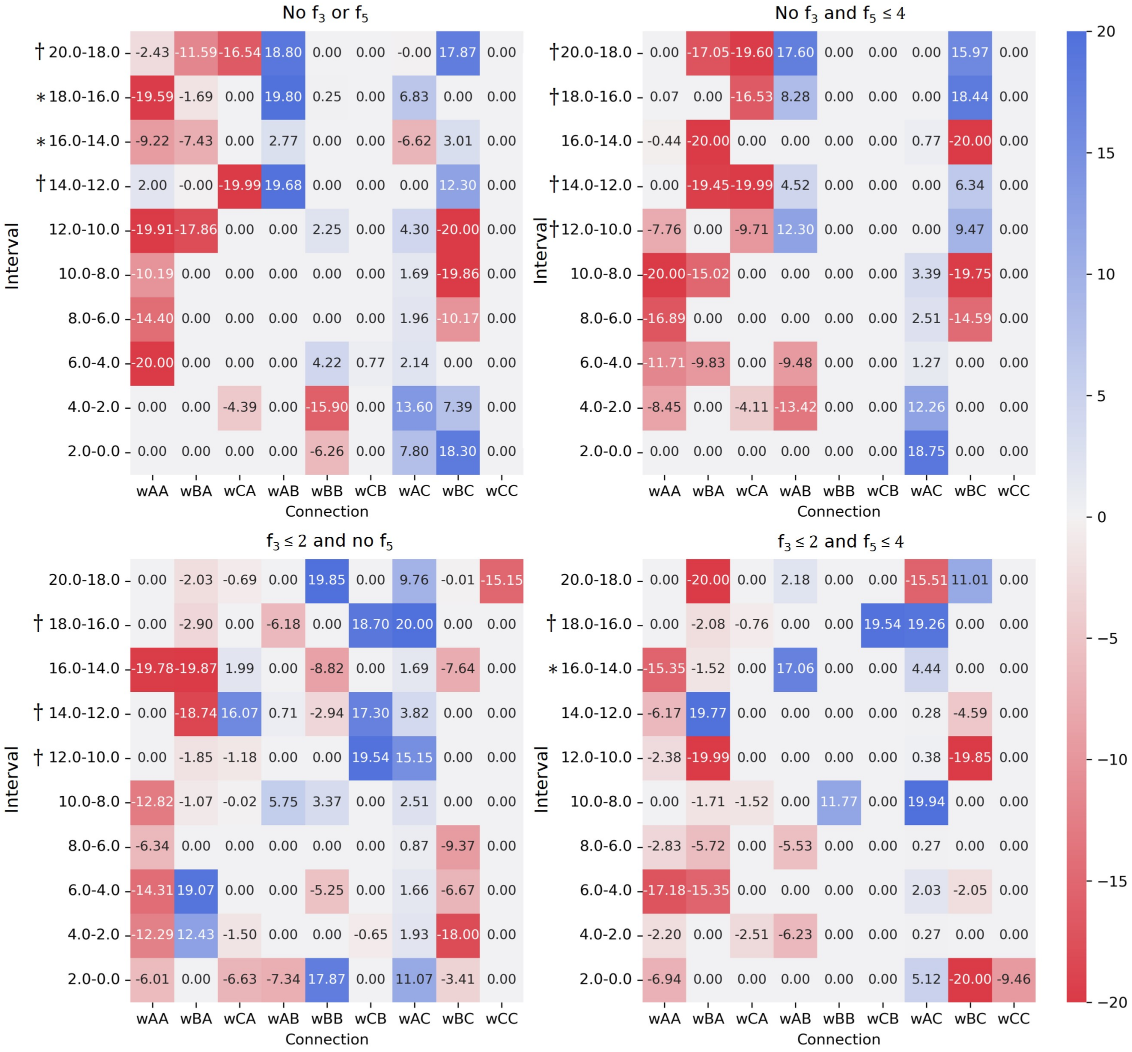

Figure S6: A collection of heatmaps of the FFL node connections as found by the FFL optimisation. By displaying the node connections for each boundary in a heatmap, it was hypothesised that a general design of an FFL showing dose-response behaviour with a sharp switch could be inferred. One row in one heatmap shows all the node connections of the FFL optimised within the specified f_4 interval. Blue values indicate activation, while red values indicate inhibition. Each heatmap shows a different combination of constraints f_3 and f_5. Solutions that cause oscillations are marked by a dagger and the three best solutions are marked by an asterisk. These visualisations were made with Seaborn and Matplotlib in Jupyter Notebook version 6.3.0 using Python 3.12.3.

S2.2 TMSD-F

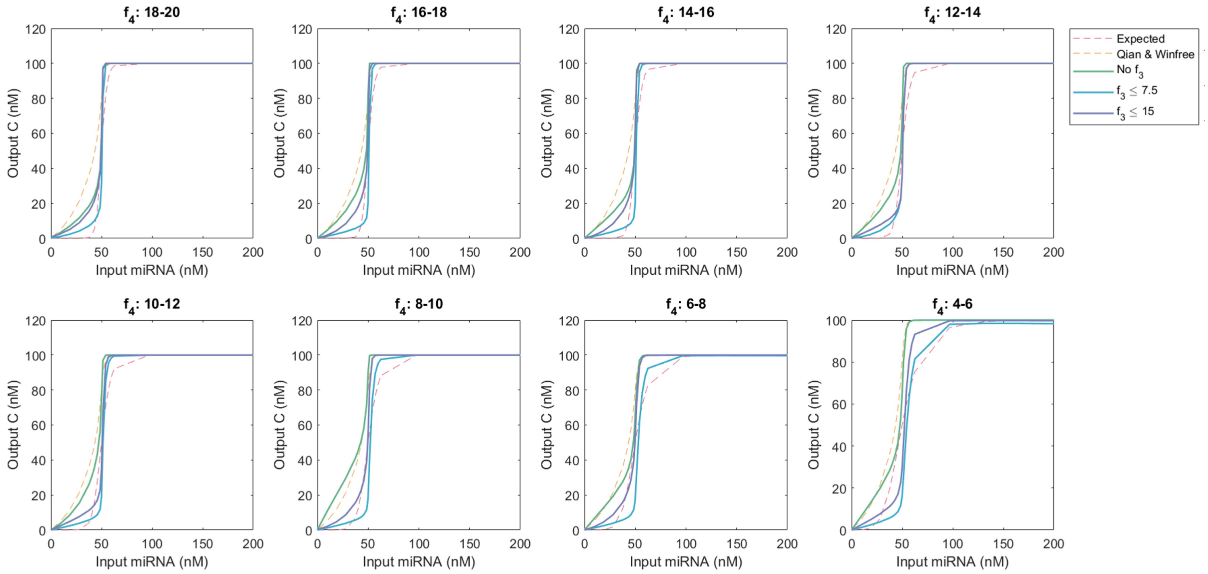

Figure S7: Comparative dose-response curves from the TMSD-F optimisation. Each subplot shows a slope (f_2) constraint interval. Expected curves are computed with the Hill function (intercept = 0.01, maximum output = 100 and slope = average of the interval) and plotted in pink. The Qian & Winfree solution is plotted with the given rates in orange [6]. The other solutions are coloured according to their additional constraints on f_3. $K_{expected}$ was set to 50 nM.

Table S3: Rate values for the TMSD-F system as found by the multi-objective optimisation and the values from Qian & Winfree (2011).

**Rate**	**Qian & Winfree (2011)**	**Optimised**
k_f	$2 \times 10^6 M^{-1}s^{-1}$	$3.4 \times 10^7 M^{-1}s^{-1}$
k_s	$5 \times 10^4 M^{-1}s^{-1}$	$2.5 \times 10^4 M^{-1}s^{-1}$
k_{rf}	$10\ s^{-1}$	$32.3\ s^{-1}$
k_{rs}	$0.5\ s^{-1}$	$0.13\ s^{-1}$
k_l	$1\ M^{-1}s^{-1}$	$0.02\ M^{-1}s^{-1}$

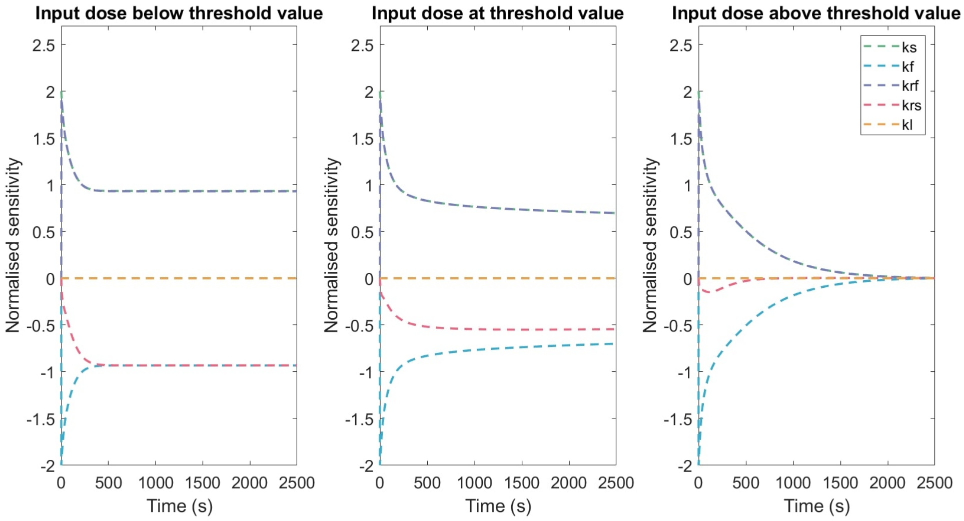

Figure S8: Parameter sensitivity analysis on the output fluorescence of the optimised TMSD-F system. The normalised sensitivities were computed for a dose below (30 nM), at (50 nM) and above (70 nM) $K_{expected}$ with $K_{expected}$ equal to 50 nM. A positive sensitivity indicates an increase in fluorescence when a respective parameter is increased, while a negative sensitivity indicates a decrease in fluorescence when a respective parameter is increased.

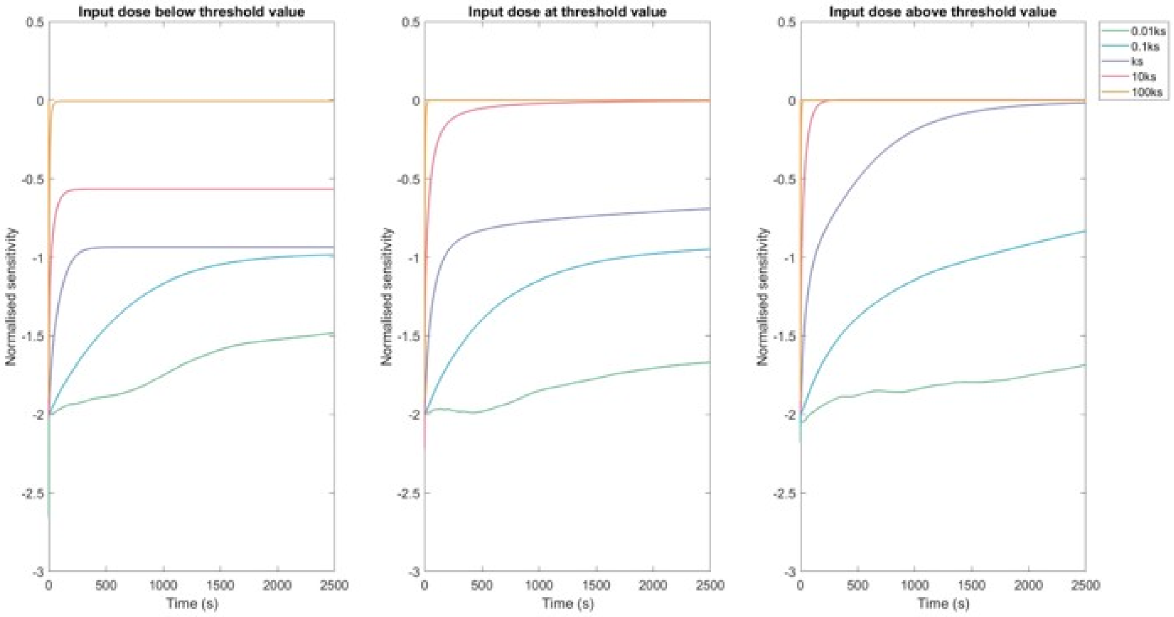

Figure S9: Parameter sensitivity analysis of k_f for different values of k_s on the output fluorescence of the optimised TMSD-F system. The normalised sensitivities were computed for a dose below (30 nM), at (50 nM) and above (70 nM) $K_{expected}$ with $K_{expected}$ equal to 50 nM.

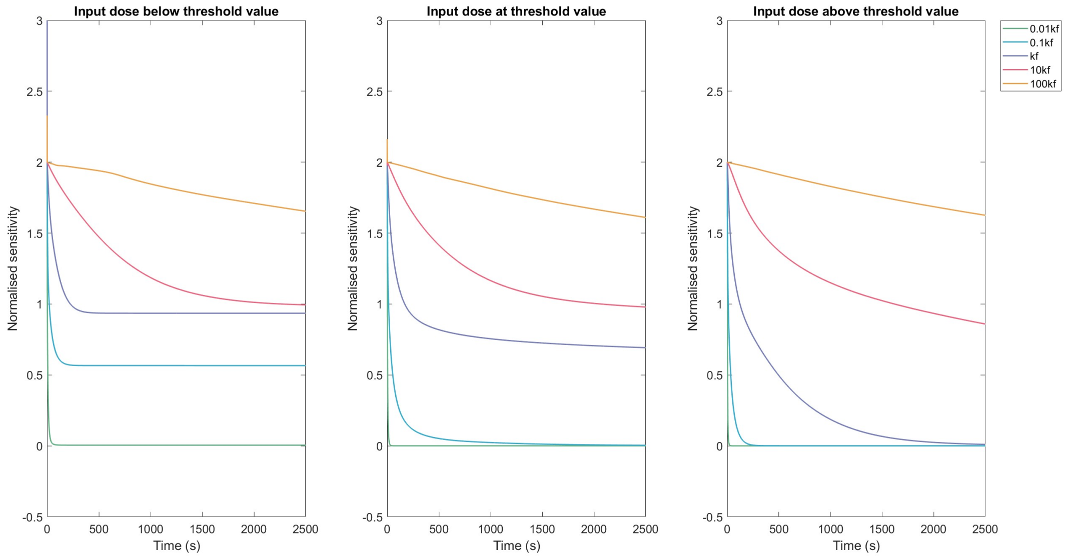

Figure S10: Parameter sensitivity analysis of k_s for different values of k_f on the output fluorescence of the optimised TMSD-F system. The normalised sensitivities were computed for a dose below (30 nM), at (50 nM) and above (70 nM) $K_{expected}$ with $K_{expected}$ equal to 50 nM.

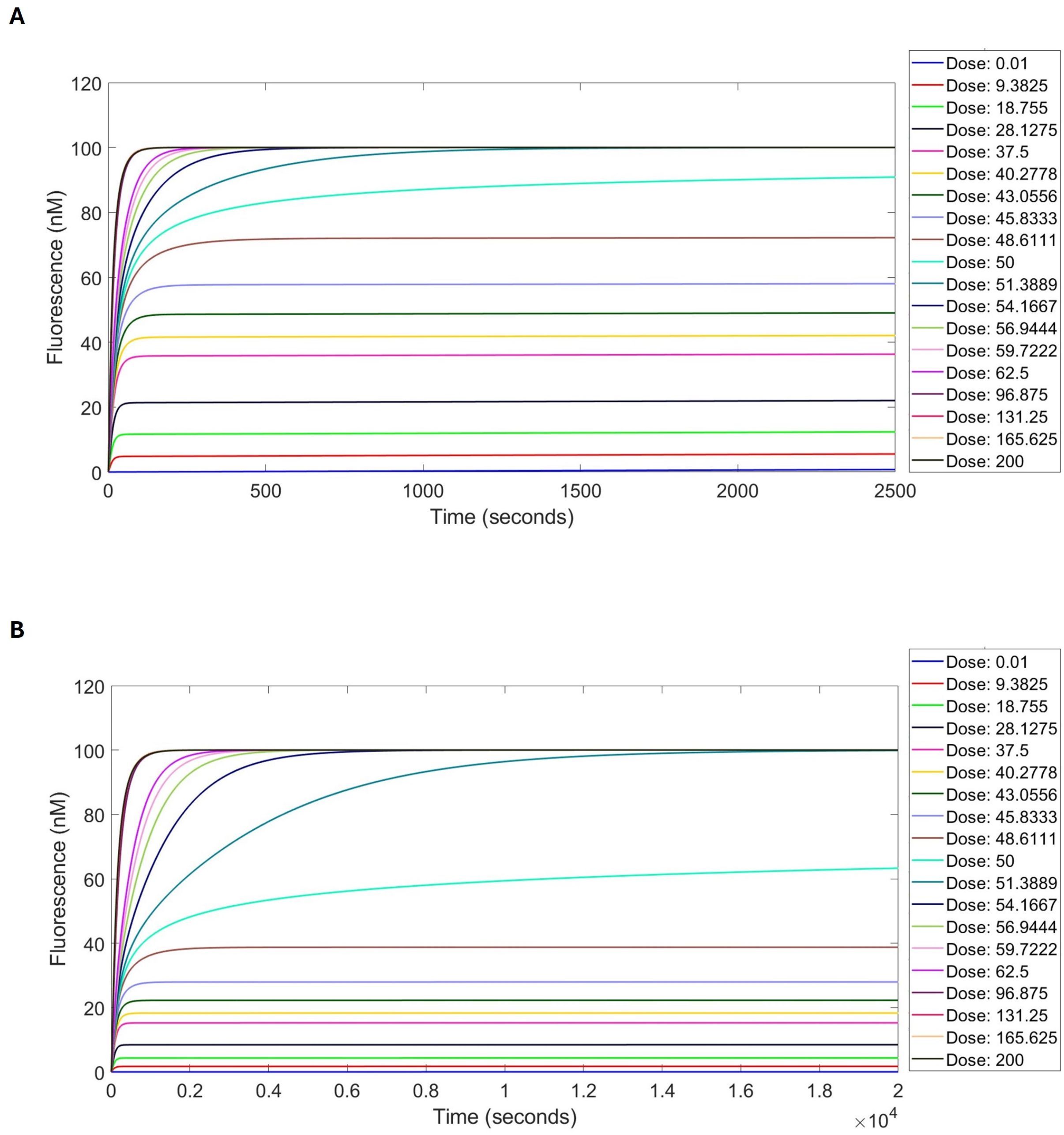

Figure S11: Time responses for the TMSD-F systems showing how long it takes to reach the steady state for each dose ($K_{expected} = 50\ nM$. A) Unoptimised TMSD-F from Qian & Winfree, which reaches steady state values after 1500 seconds. B) Best solution from the TMSD-F optimisation, which reaches steady state values for all doses after 20,000 seconds.

S2.3 TMSD-NF

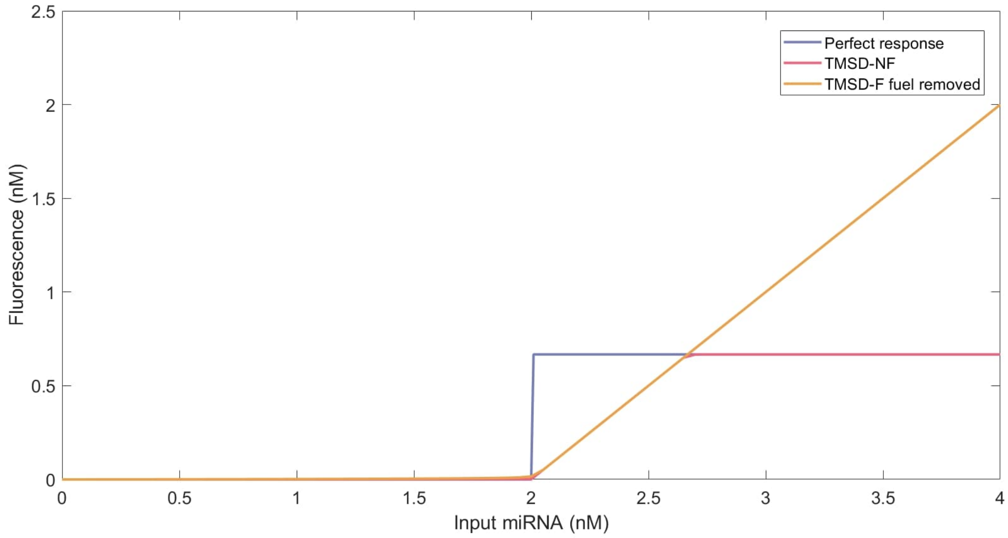

Figure S12: Dose-response curves of a perfect binary response (purple), the TMSD-NF system (pink) and the TMSD-F system without the fuel concentration set to 0, so no fuel reaction could take place (yellow). $K_{expected}$ was set to 2.

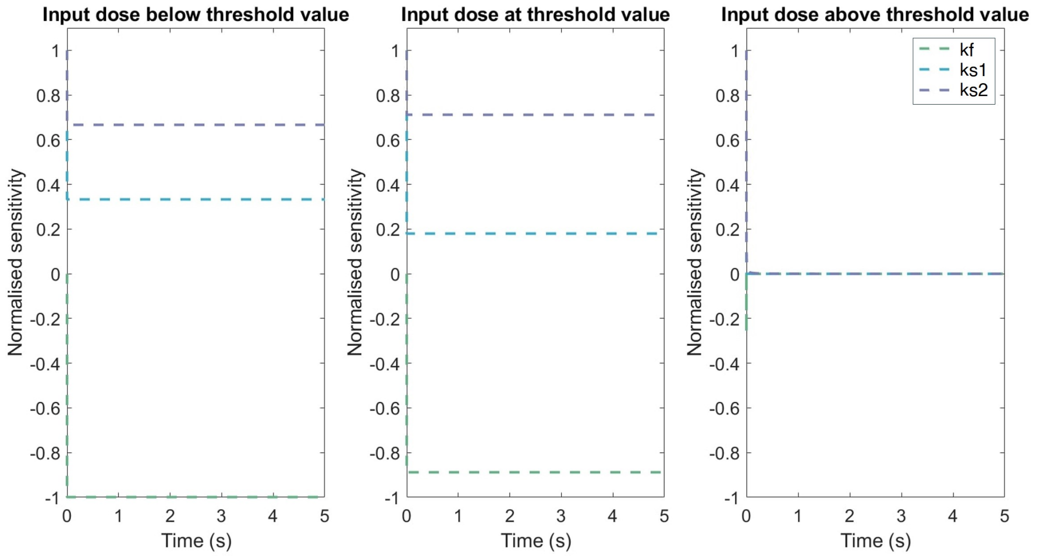

Figure S13: Parameter sensitivity analysis on the output fluorescence of the TMSD-NF system. The normalised sensitivities were computed for a dose below (1 nM), at (2 nM) and above (3 nM) $K_{expected}$ with $K_{expected}$ equal to 2 nM.

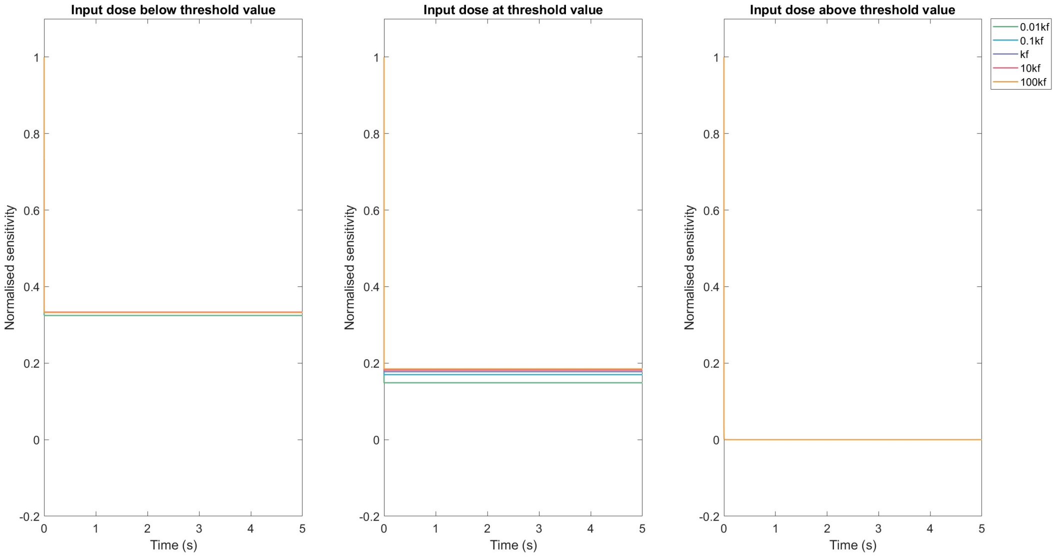

Figure S14: Parameter sensitivity analysis of k_s1 for different values of k_f on the output fluorescence of the optimised TMSD-NF system. The normalised sensitivities were computed for a dose below (1 nM), at (2 nM) and above (3 nM) $K_{expected}$ with $K_{expected}$ equal to 50 nM. The lines for $0.1k_f$, k_f, $10k_f$ and $100k_f$ overlap in the right pane.

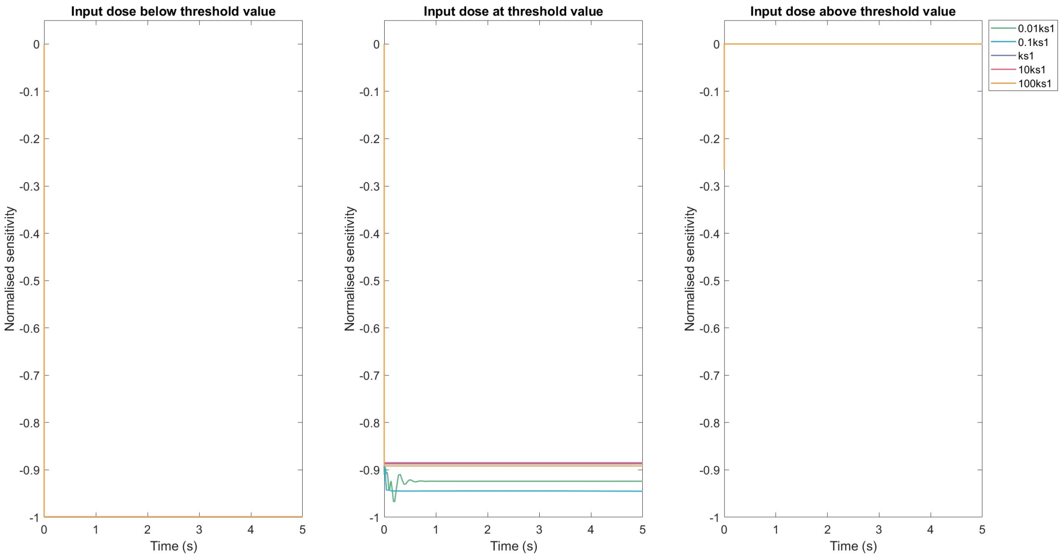

Figure S15: Parameter sensitivity analysis of k_f for different values of k_s1 on the output fluorescence of the optimised TMSD-NF system. The normalised sensitivities were computed for a dose below (1 nM), at (2 nM) and above (3 nM) $K_{expected}$ with $K_{expected}$ equal to 50 nM.

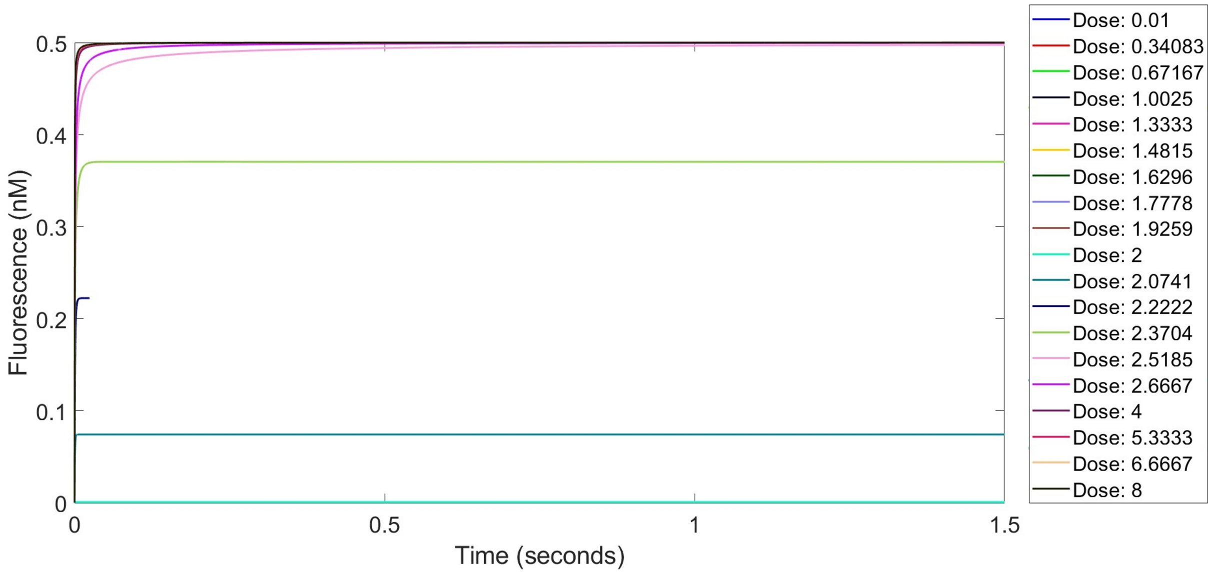

Figure S16: Time responses for the TMSD-NF system showing how long it takes to reach the steady state for each dose.

S2.3.1 Determining the best ratio of initial concentrations

The threshold mechanism should respond with the same shape of dose-response curve if the $K_{expected}$ is changed. This feature is important for future miRNA tests, as each miRNA will have a different $K_{expected}$. The initial conditions can be controlled in the lab and can regulate the shape of the curve. The initial concentrations of the TMSD-F system are based on the $K_{expected}$, making sure the same ratio between the initial conditions is used, which translates to the same shape of the dose-response curve for every $K_{expected}$ (Figure S17 orange). The TMSD-NF system did not yet scale for $K_{expected}$ and thus creates different dose-response curves for different $K_{expected}$ values (Figure S17 pink). The higher the $K_{expected}$ compared to the concentrations of the reporters IO and P, the sharper the switch seems. A qualitative analysis of the TMSD-NF system was performed to find the optimal ratio of input concentrations to achieve the same curve shape every time. Smaller concentrations were used to better match ongoing lab work at the time.

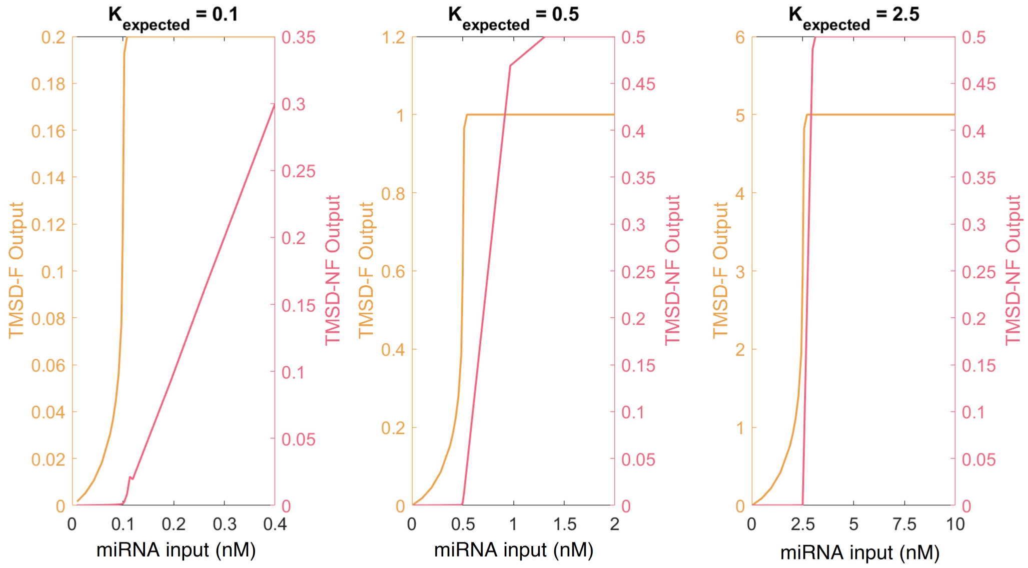

Figure S17: Comparison of TMSD-F (orange) and TMSD-NF (red) in producing dose-response curves for different threshold values. The curves were simulated for three different threshold values (0.1, 0.5, 2.5). In TMSD-F, the ratio is as follows: C1: $2K_{expected}$; F: $4K_{expected}$; TH: $K_{expected}$ and R: $3K_{expected}$. In TMSD-NF, the concentration of IO and P are kept constant at 0.5. The TMSD-F curves have a constant shape, while the TMSD-NF curve improves at higher threshold values.

The concentrations of IO and P are made dependent on the concentration of unbound TH, which determines $K_{expected}$, to resemble the scaling in the TMSD-F system. So, $IO = P = \frac{TH}{r}$, where r is iteratively changed in the analysis to find the best dose-response curve. The output must be kept as high as possible while maintaining a steep switch in the curve. An r equal to 3 presented as the best trade-off between these two objectives (Figure S18). Thus, for a functioning TMSD-NF system, three times as little IO and P compared to TH was deemed necessary.

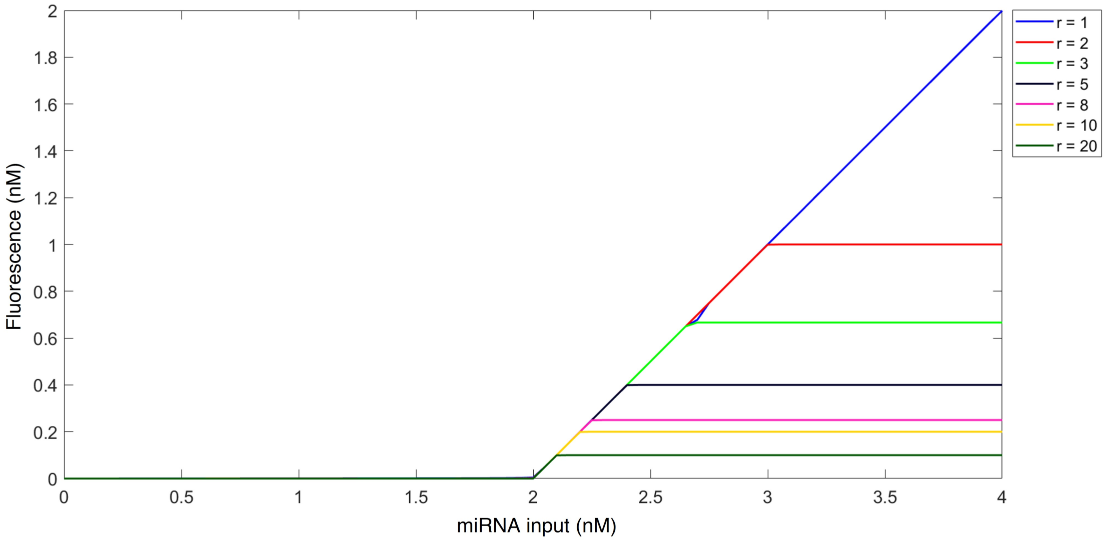

Figure S18: Simulated dose-response curves of $K_{expected} = 2$ for varying levels of r. Lower concentrations of IO and P (caused by higher values of r), result in a sharper dose-response curve and a lower maximum output.

References

[1] Jordan Ang et al. “Tuning Response Curves for Synthetic Biology”. In: *ACS Synthetic Biology* 2.10 (Oct. 2013), pp. 547–567. DOI: 10.1021/sb4000564. URL: https://doi.org/10.1021/sb4000564 (visited on 04/26/2024).

[2] J. W. Hearne. “Sensitivity analysis of parameter combinations”. In: *Applied Mathematical Modelling* 9.2 (Apr. 1985), pp. 106–108. ISSN: 0307-904X. DOI: 10.1016/0307-904X(85)90121-0. URL: https://www.sciencedirect.com/science/article/pii/0307904X85901210 (visited on 11/06/2024).

[3] Robert R. F. Machinek et al. “Programmable energy landscapes for kinetic control of DNA strand displacement”. In: *Nature Communications* 5.1 (Nov. 2014), p. 5324. ISSN: 2041-1723. DOI: 10.1038/ncomms6324. URL: https://www.nature.com/articles/ncomms6324 (visited on 07/04/2024).

[4] “miRADAR: WUR 2024 iGEM”. In: (2024). DOI: 2024.igem.wiki/wageningenur/. URL: https://2024.igem.wiki/wageningenur/ (visited on 11/26/2024).

[5] Irene Otero-Muras and Julio R. Banga. “Automated Design Framework for Synthetic Biology Exploiting Pareto Optimality”. In: *ACS Synthetic Biology* 6.7 (July 2017), pp. 1180–1193. ISSN: 2161-5063, 2161-5063. DOI: 10.1021/acssynbio.6b00306. URL: https://pubs.acs.org/doi/10.1021/acssynbio.6b00306 (visited on 04/14/2024).

[6] Lulu Qian and Erik Winfree. “Scaling Up Digital Circuit Computation with DNA Strand Displacement Cascades”. In: *Science* 332.6034 (June 2011), pp. 1196–1201. DOI: 10.1126/science.1200520. URL: https://www.science.org/doi/10.1126/science.1200520 (visited on 07/04/2024).

[7] David Yu Zhang and Erik Winfree. “Control of DNA Strand Displacement Kinetics Using Toehold Exchange”. In: *Journal of the American Chemical Society* 131.47 (Dec. 2009), pp. 17303–17314. ISSN: 0002-7863. DOI: 10.1021/ja906987s. URL: https://doi.org/10.1021/ja906987s (visited on 06/19/2024).
